# Occupational Exposure to Silica Dust and Silicosis Risk in Chinese Noncoal Mines: Qualitative and Quantitative Risk Assessment

**DOI:** 10.2196/56283

**Published:** 2024-09-02

**Authors:** Kai Liu, Xin Sun, Wei-Jiang Hu, Liang-Ying Mei, Heng-Dong Zhang, Shi-Biao Su, Kang Ning, Yun-Feng Nie, Le-Ping Qiu, Ying Xia, Lei Han, Qiang Zhi, Chun-Bo Shi, Geng Wang, Wei Wen, Jian-Qiong Gao, Bing Yu, Xin Wang, Yi-Wen Dong, Ning Kang, Feng Han, Hong-Ying Bian, Yong-Qing Chen, Meng Ye

**Affiliations:** 1 National Institute for Occupational Health and Poison Control Chinese Center for Disease Control and Prevention Beijing China; 2 Institute of Occupational Disease Prevention Hubei Provincial Center for Disease Control and Prevention Wuhan China; 3 Institute of Occupational Disease Prevention Jiangsu Provincial Center for Disease Control and Prevention Nanjing China; 4 Institute of Occupational Health Assessment Guangdong Province Hospital for Occupational Disease Prevention and Treatment Guangzhou China; 5 Institute of Occupational Disease Prevention Liaoning Provincial Center for Disease Control and Prevention Shenyang China; 6 Department of Health Risk Assessment Hunan Prevention and Treatment Institute for Occupational Diseases Changsha China; 7 Institute of Occupational Health and Radiological Health Sichuan Center for Disease Control and Prevention Chengdu China; 8 Department of Occupational Disease Prevention and Control Inner Mongolia Autonomous Region Center for Disease Control and Prevention Hohhot China; 9 Institute of Occupational Health and Public Health Qinghai Center for Disease Control and Prevention Xining China; 10 Enshi Tujia and Miao Autonomous Prefectural Center for Disease Control and Prevention Enshi China

**Keywords:** Chinese noncoal mine, silica dust, silicosis, qualitative risk assessment, quantitative risk assessment, qualitative and quantitative risk assessment

## Abstract

**Background:**

Despite increasing awareness, silica dust–induced silicosis still contributes to the huge disease burden in China. Worryingly, recent silica dust exposure levels and silicosis risk in Chinese noncoal mines remain unclear.

**Objective:**

We aimed to determine recent silica dust exposure levels and assess the risk of silicosis in Chinese noncoal mines.

**Methods:**

Between May and December 2020, we conducted a retrospective cohort study on 3 noncoal mines and 1 public hospital to establish, using multivariable Cox regression analyses, prediction formulas of the silicosis cumulative hazard ratio (H) and incidence (I) and a cross-sectional study on 155 noncoal mines in 10 Chinese provinces to determine the prevalence of silica dust exposure (PDE), free silica content, and total dust and respirable dust concentrations. The qualitative risk of silicosis was assessed using the International Mining and Metals Commission’s risk-rating table and the occupational hazard risk index; the quantitative risk was assessed using prediction formulas.

**Results:**

Kaplan-Meier survival analysis revealed significant differences in the silicosis probability between silica dust–exposed male and female miners (log-rank test χ21=7.52, *P*=.01). A total of 126 noncoal mines, with 29,835 miners and 4623 dust samples, were included; 13,037 (43.7%) miners were exposed to silica dust, of which 12,952 (99.3%) were male. The median PDE, free silica content, total dust concentration, and respirable dust concentration were 61.6%, 27.6%, 1.30 mg/m3, and 0.58 mg/m3, respectively, indicating that miners in nonmetal, nonferrous metal, small, and open-pit mines suffer high-level exposure to silica dust. Comprehensive qualitative risk assessment showed noncoal miners had a medium risk of silicosis, and the risks caused by total silica dust and respirable silica dust exposure were high and medium, respectively. When predicting H and I over the next 10, 20, and 30 years, we assumed that the miner gender was male. Under exposure to current total silica dust concentrations, median I10, I20, and I30 would be 6.8%, 25.1%, and 49.9%, respectively. Under exposure to current respirable silica dust concentrations, median I10, I20, and I30 would be 6.8%, 27.7%, and 57.4%, respectively. These findings showed that miners in nonmetal, nonferrous metal, small, and open-pit mines have a higher I and higher qualitative silicosis risk.

**Conclusions:**

Chinese noncoal miners, especially those in nonmetal, nonferrous metal, small, and open-pit mines, still suffer high-level exposure to silica dust and a medium-level risk of silicosis. Data of both total silica dust and respirable silica dust are vital for occupational health risk assessment in order to devise effective control measures to reduce noncoal mine silica dust levels, improve miners’ working environment, and reduce the risk of silicosis.

## Introduction

Silica, especially crystalline silica, has long been recognized as a common and serious hazard in a variety of industrial activities in the workplace worldwide. Occupational silica exposure is entirely preventable through interventions, such as wet cutting, good ventilation, and well-fitted supplied-air respirators [1]. Despite increasing awareness of silica toxicity [2,3] and silicosis pathophysiology [4], recent studies show there are still a large number of workers exposed to this mineral dust worldwide [1,5-7]. In addition, the burden of silica-related diseases remains serious [8,9]. The outbreaks of silicosis [10-12] and other silica-related diseases [13-15] have caused great concern over occupational exposure to silica dust, especially in younger workers [16,17] and low- and middle-income countries [4]. In 1995, the International Labour Organization (ILO)/World Health Organization (WHO) joint committee on occupational health established the ILO/WHO global program for elimination of silicosis from the world by 2030. Unfortunately, the campaign does not appear to have maintained momentum, and newly diagnosed pneumoconiosis cases have increased on a global scale in recent decades [18].

Occupational health in China started in the 1950s. In the past decades, the government has made various efforts to improve occupational health, such as the National Basic Public Health Service Program and Action Plan for the Prevention and Control of Pneumoconiosis (of which silicosis is the most prevalent type) [19-21]. From 1950 to 2003, the annual mean respirable (silica) dust concentration showed a gradual decline in Chinese noncoal mines, and the concentration fell to less than 0.1 mg/m^3^ after 1970 because of increased protective measures [22]. However, from 1949 to 2019, the number of pneumoconiosis cases and deaths in Hubei Province (China) increased [23]. At the end of 2018, the total number of reported occupational cases was up to 975,000, and 90% of them were pneumoconiosis, which was mainly distributed in the mining industry and younger workers [24,25]. At a global level, China was 1 of the nations most affected by the burden of silicosis, accounting for 88.3% of new pneumoconiosis cases, and had the highest age-standardized rates of incidence and prevalence in 2019 [26-28]. At present, the booming mining industry in China might place large numbers of workers at risk [1], and occupational silica dust exposure remains a potential challenge in improving Chinese occupational health, especially in noncoal mines [4,29]. Noncoal mines, defined as mines other than coal mines, mainly include metal and nonmetal mines [30]. In 2012, more than 23 million workers were exposed to silica dust in China [22,26]. By February 2022, there were about 37,000 noncoal mine enterprises in China, most of which were lagging in technology and equipment [31,32]. Therefore, it is necessary to determine the current dust exposure and silicosis risk levels in Chinese noncoal mines.

In this study, we undertook a retrospective cohort investigation into the link between silica dust exposure and the quantitative risk of silicosis in 3 noncoal mines and 1 public hospital. A cross-sectional study was also conducted based on 155 noncoal mines selected from 10 Chinese provincial regions in which noncoal mines are concentrated. Using qualitative and quantitative assessment methods, the risk of silicosis in Chinese noncoal mines was assessed from different perspectives: mine categories, production scales, mining methods, and jobs. The findings will provide crucial evidence for improving occupational health and fulfilling the promise of a healthy China.

## Methods

### Study Design

Between May and December 2020, we conducted a retrospective cohort study and a cross-sectional study based on noncoal mines and assessed the qualitative and quantitative risks of silicosis. The retrospective cohort study, carried out on 3 noncoal mines and 1 public hospital, was designed to establish formulas for predicting the silicosis cumulative hazard ratio (HR; H) and incidence (I) in noncoal mines. The cross-sectional study, carried out on 155 noncoal mines, was designed to investigate recent levels of silica dust exposure in Chinese noncoal mines. Due to the uneven geographical distribution of mineral resources, nonprobability convenience-based sampling was used to select the 155 noncoal mines from 10 provinces in northern, western, southern, eastern, and central China. The inclusion criteria for silica dust–exposed noncoal mines were as follows: (1) nonferrous metal, ferrous metal, or nonmetal (except fuels) mines; (2) 10%≤free silica content≤100%; (3) complete basic information about noncoal mines; and (4) no extremely abnormal determination result(s). The term “exposure” used in this study was defined as the concentration (mg/m^3^) of airborne occupational dust that was measured in workers’ upper anterior chest area or breathing zone (about 1 foot from the mouth or nose), and the term “job” was defined as a collection of types of work requiring similar skills, responsibilities, training, and process of production [33].

### Data Collection

In this retrospective cohort study, monitoring data, collected from 1940 to 2020, included 2 parts: (1) silica dust–exposed workers’ jobs and occupational health records and (2) the free silica content and silica dust concentrations the workers were exposed to in their jobs. Occupational health records included initial (first) and end (last) dates of silica dust exposure, chest X-ray examination results, and the date of initial diagnosis. Silicosis was diagnosed according to each worker’s chest X-ray examination, occupational exposure history, clinical manifestation, and workplace environment measurement. All workers with silicosis were followed up until the date of initial diagnosis. Monitoring data of the silica dust concentration were used to create a job exposure matrix (JEM). Since the 1950s, the Chinese government has enforced systematic dust-sampling regulations, which require mines to measure dust concentrations monthly in dusty work areas and measure the silica content in bulk dust, if necessary [34]. The respirable dust–sampling regulations were implemented in 1992. Therefore, before 1992, the respirable dust concentration was estimated based on the contemporaneous monitoring of the total dust concentration of the same or a similar job. The formula of respirable dust concentration estimation was *0.30 × total dust concentration of the same or similar job + 0.08*, where the coefficient of determination (*R*^2^)=0.94. Both total dust and respirable dust concentrations were measured as 8-hour time-weighted average concentrations (C_TWA_, mg/m^3^) using gravimetric samplers and microbalance:



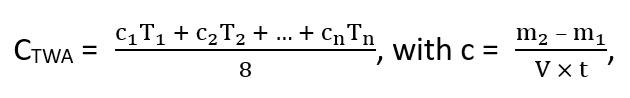



,where c is the dust concentration (mg/m^3^), m_2_ is the postsample filter weight (mg), m_1_ is the presample filter weight (mg), V is the average sample flow rate (L/minute), t is the sampling duration (minutes), and T is the working hours under exposure to different concentrations of dust. The free silica content (%) was determined using the pyrophosphate method:



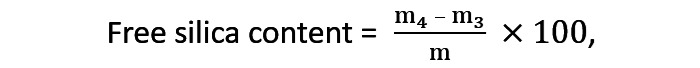



where m_4_ is the crucible and free silica weight (g), m_3_ is the crucible weight (g), and m is the bulk dust weight (g).

Regarding survey data for this cross-sectional study, the basic information about noncoal mines included the mining method, mine category, product type, production scale [35], number of workers, and prevalence of silica dust exposure (PDE). We carried out 3-day continuous sampling determination once a day, and 3 samples were taken each time from the same sampled site: 1 air dust sample for total dust concentration measurement, 1 air dust sample for respirable dust concentration measurement, and 1 bulk dust sample for free silica content determination. Overall, 9 dust samples were collected for each job during the 3-day sampling period. Air dust samples at the mobile and fixed work sites, such as the inspector and the crusher, were separately collected via personal sampling and area sampling. Personal sampling dust samples at the mobile work site were collected from workers’ upper anterior chest area using air sampling pumps at an air flow rate of 1-5 L/minute. Area sampling dust samples at the fixed work site were collected from workers’ breathing zone (near the collar) using air sampling pumps at an air flow rate of 5-80 L/minute. In addition, to make sure the highest and lowest dust concentrations were included, the sample time of area sampling per day was ≥45 minutes, which was divided into 3 intermittent sample times (≥15 minutes each time). All on-site measurements were performed on days with no rain, and calibration of the air sampler was performed before and after each use. Dust concentrations were gravimetrically analyzed using an XP105DR Microbalance (METTLER TOLEDO Inc). The free silica content in the dustfall sample (bulk dust) was determined using the pyrophosphate method. Total silica dust concentrations were calculated by multiplying total dust concentrations by the free silica content, and respirable silica dust concentrations were calculated by multiplying respirable dust concentrations by the free silica content.

### Statistical Analysis

Statistical analysis consisted of 3 parts. First, we established prediction formulas for the silicosis cumulative HR (H) and incidence (I). Kaplan-Meier survival analysis and multivariable Cox regression analysis were conducted to establish the formulas based on cumulative exposure to total silica dust (D_t_) and cumulative exposure to respirable silica dust (D_r_). The male gender was coded as 1, and the female gender worker was coded as 0.

Second, we determined Chinese noncoal mines’ characteristics and silica dust exposure levels. To estimate the overlimit degree of silica dust exposure, we calculated the percentages of dust sample (concentrations) exceeding China’s occupational exposure limit (OEL) or exceeding the exposure limit recommended by the American Conference for Governmental Industrial Hygienists (ACGIH; recommended exposure limit [REL] of respirable silica dust=0.025 mg/m^3^).

**Figure 1:**
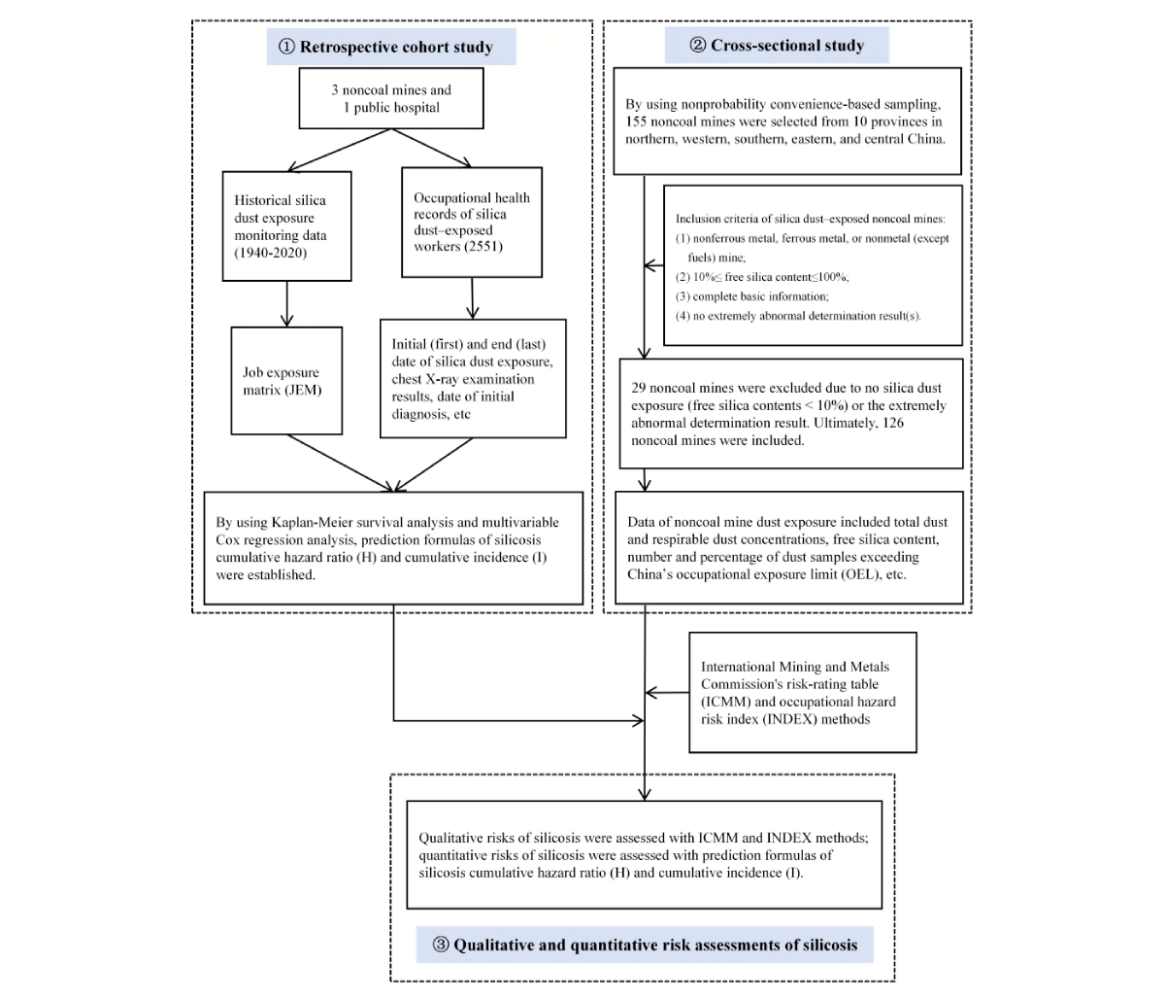
Flowchart of qualitative and quantitative silicosis risk assessments in Chinese noncoal mines. First, a retrospective cohort study was designed to establish prediction formulas of the silicosis cumulative HR (H) and silicosis cumulative incidence (I). Second, using nonprobability convenience-based sampling, 155 noncoal mines were selected from 10 provinces in northern, western, southern, eastern, and central China. Air dust samples at the mobile and fixed work sites were separately collected via personal sampling and area sampling. Third, the qualitative risk of silicosis was assessed using the ICMM and INDEX, and the quantitative risk was assessed using the prediction formulas. HR: hazard ratio; ICMM: International Mining and Metals Commission's risk-rating table; INDEX: occupational hazard risk index.

Finally, we conducted qualitative and quantitative silicosis risk assessments of Chinese noncoal miners. The International Mining and Metals Commission's risk-rating table (ICMM) and the occupational hazard risk index (INDEX) were used in qualitative assessment [36-39], and prediction formulas of the silicosis cumulative HR (H) and incidence (I) were used in quantitative risk assessment (Figure 1). There are 5 kinds of qualitative risk levels for both the ICMM and INDEX: tolerable risk/no hazard, potential risk/mild hazard, high risk/moderate hazard, very high risk/severe hazard, and intolerable risk/extreme hazard. To assess the comprehensive qualitative risk of noncoal mines, each characteristic’s weighted score (S_weighted_) was calculated based on the percentage of dust samples in the raw 5-level risks, separately coded as 1, 2, 3, 4, and 5:



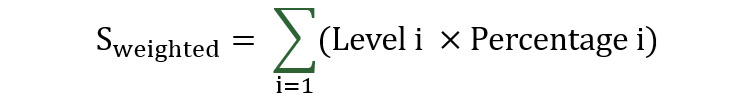



The comprehensive qualitative risk level was classified as very low risk (0≤S_weighted_≤1), low risk (1.01≤S_weighted_≤2.00), medium risk (2.01≤S_weighted_≤3.00), high risk (3.01≤S_weighted_≤4.00), and very high risk (4.01≤S_weighted_≤5.00). In addition, if S_weighted_ of both the ICMM and INDEX was ≥3.01 or S_weighted_ of either was ≥4.01, the comprehensive risk level was identified as high. Continuous data of dust exposure and silicosis risk were expressed as the median (IQR).

The OEL for dust exposure depends on the free silica content: if 10%≤free silica content≤50%, OELs for total dust and respirable dust are 1 and 0.7 mg/m^3^, respectively; if 50%<free silica content≤80%, OELs are 0.7 and 0.3 mg/m^3^, respectively; and if free silica content>80%, OELs are 0.5 and 0.2 mg/m^3^, respectively. All statistical analyses were conducted using SAS version 9.4 (SAS Institute).

### Ethical Considerations

The Medical Ethical Review Committee of the National Institute for Occupational Health and Poison Control, Chinese Center for Disease Control and Prevention, approved this study (approval number 201811), with the requirement for informed consent being waived. In this study, there was no monetary or nonmonetary compensation provided to participants. No identification of individual participants was shown in any figures or multimedia appendices. Access to worker- and mine-identifiable data was restricted to authorized individuals and was only accessible on 2 designated computers.

## Results

### Prediction Formulas of Silicosis Cumulative Hazard Ratios and Incidences

In this retrospective cohort study, a total of 2551 silica dust–exposed workers were selected from 3 noncoal mines, including 1 large and 2 medium-size mines. Monitoring data showed that the C_TWA_ of total dust ranged from 0.06 to 41.94 mg/m^3^, of respirable dust ranged from 0.05 to 12.54 mg/m^3^, and of free silica content ranged from 16.8% to 17.3%. Of the 2551 workers, 1512 (59.3%) were still working in the enterprises, while the other 1039 (40.7%) had left, retired, or died. In addition, their follow-up time ranged from 1 year to 68 years, and the total follow-up time was up to 57,480 person-years. By December 2020, 247 (16.3%) workers were diagnosed with silicosis, 221 (89.5%) of whom had stage I silicosis at the initial diagnosis (see Table 1 and Table S1 in Multimedia Appendix 1).

**Figure 2:**
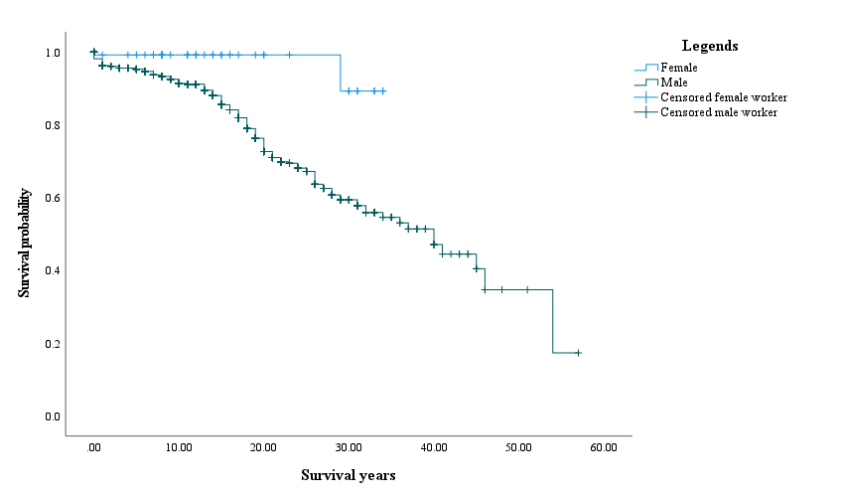
Kaplan-Meier survival curves of silica dust–exposed noncoal miners. The x-axis and y-axis represent survival years and the cumulative survival probability, respectively. Cross short lines refer to censoring, and vertical longer lines pointing downward refer to occurrence of events.

Kaplan-Meier survival analysis showed that there were significant differences in the silicosis probability between silica dust–exposed male and female miners (log-rank test *χ*^2^_1_=7.52, *P*=.01), as shown in Figure 2, but there was no difference among different mine categories, the production scale, mining methods, the initial silica dust exposure age, the initial silica dust exposure year, or smoking history.

Regarding total silica dust exposure, multivariable Cox regression analysis showed that gender (hazard ratio [HR]=4.96, β=1.60, *P*=.02) and cumulative exposure to total silica dust (HR=1.07, β=0.07, *P*<.001) were independent predictors of silicosis morbidity in noncoal miners. Over the next 10, 20, and 30 years, prediction formulas of the silicosis cumulative HR (H) caused by total silica exposure would be as follows:

H_10_ = 0.09 × exp(1.60[gender – 0.95] + 0.07[D_t_ – 8.01])

H_20_ = 0.27 × exp(1.60[gender – 0.95] + 0.07[*D*_t_ – 8.01])

H_30_ = 0.50 × exp(1.60[gender – 0.95] + 0.07[D_t_ – 8.01])

The prediction formulas of the silicosis cumulative incidence (I) would be as follows:

I_10_ = 1 – 0.92^exp(1.60[gender – 0.95] + 0.07[Dt – 8.01])^

I_20_ = 1 – 0.76^exp(1.60[gender – 0.95] + 0.07[Dt – 8.01])^

I_30_ = 1 – 0.61^exp(1.60[gender – 0.95] + 0.07[Dt – 8.01])^

Regarding respirable silica dust exposure, gender (HR=4.96, β=1.58, *P*=.03) and cumulative exposure to respirable silica dust (HR=1.27, β=0.24, *P*<.001) were also independent predictors of silicosis morbidity in noncoal miners. Over the next 10, 20, and 30 years, the prediction formulas of H caused by respirable silica dust exposure would be as follows:

H_10_ = 0.09 × exp(1.58[gender – 0.95] + 0.24[D_r_ – 2.64])

H_20_ = 0.27 × exp(1.58[gender – 0.95] + 0.24[D_r_ – 2.64])

H_30_ = 0.50 × exp(1.58[gender – 0.95] + 0.24[D_r_ – 2.64])

The prediction formulas of I would be as follows:

I_10_ = 1 – 0.92^exp(1.58[gender – 0.95] + 0.24[Dr – 2.64])^

I_20_ = 1 – 0.76^exp(1.58[gender – 0.95] + 0.24[Dr – 2.64])^

I_30_ = 1 – 0.61^exp(1.58[gender – 0.95] + 0.24[Dr – 2.64])^

**Table 1:** Cumulative silica dust exposure and number of silicosis cases in 3 noncoal mines.

Total silica dust					Respirable silica dust			
D_t_^a^ (mg/m^3^-years)	L_χ_^b^	W_χ_^c^	D_χ_^d^	D_r_^e^ (mg/m^3^-years)		L_χ_	W_χ_	D_χ_
≤2.46	2551	221	7	≤0.77		2551	193	6
2.46-4.92	2323	290	24	0.77-1.54		2352	269	24
4.92-7.38	2009	578	47	1.54-2.31		2059	515	39
7.38-9.84	1384	570	59	2.31-3.08		1505	595	54
9.84-12.30	755	388	60	3.08-3.85		856	421	72
12.30-14.76	307	124	34	3.85-4.62		363	160	39
14.76-17.22	149	63	10	4.62-5.39		164	74	7
17.22-19.68	76	25	3	5.39-6.16		83	33	3
19.68-22.14	48	11	1	6.16-6.93		47	13	1
22.14-24.60	36	14	1	6.93-7.70		33	13	1
24.60-27.06	21	10	1	7.70-8.47		19	10	1
27.06-29.52	10	7	0	8.47-9.24		8	6	0
29.52-31.98	3	2	0	9.24-10.01		2	1	0
>31.98	1	1	0	10.01-10.78		1	1	0

^a^D_t_: cumulative exposure to total silica dust.

^b^L_χ_: number of initial participants.

^c^W_χ_: number of participants lost to follow-up or terminated due to end of follow-up in December 2020.

^d^D_χ_: number of silicosis cases.

^e^D_r_: cumulative exposure to respirable silica dust.

### Characteristics and Silica Dust Exposure Levels of the 126 Noncoal Mines Included

**Figure 3:**
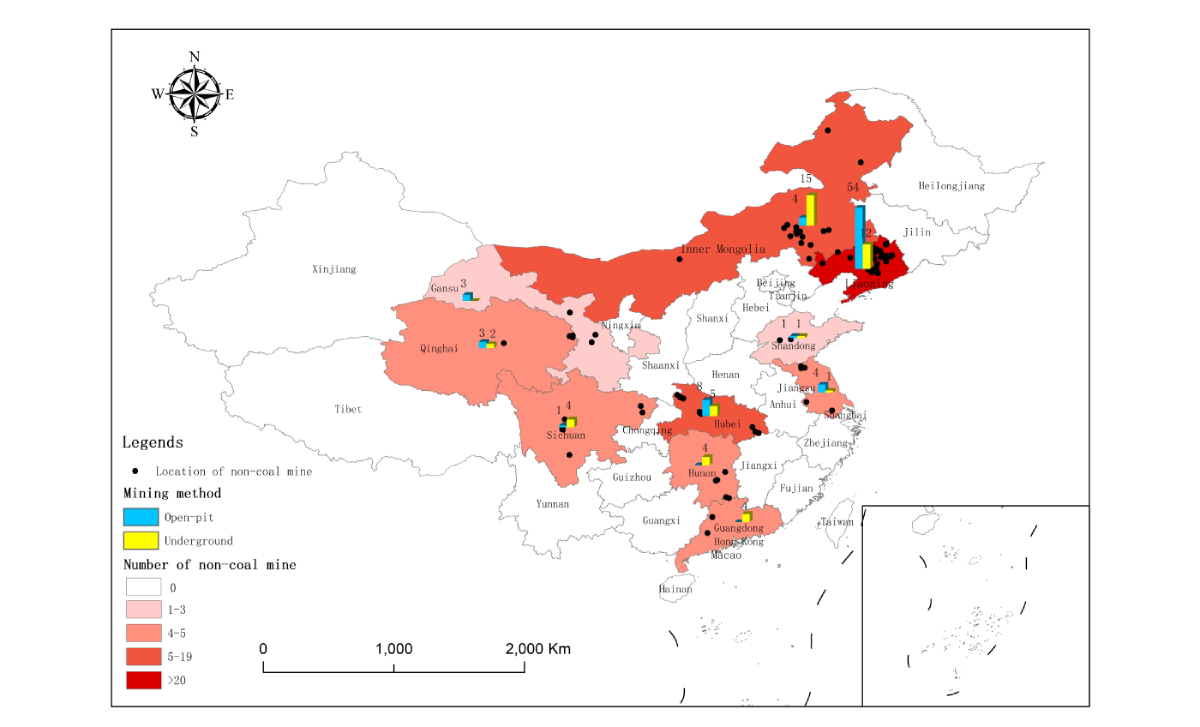
Location and mining method distribution in the 126 noncoal mines included from 10 provinces.

**Figure 4:**
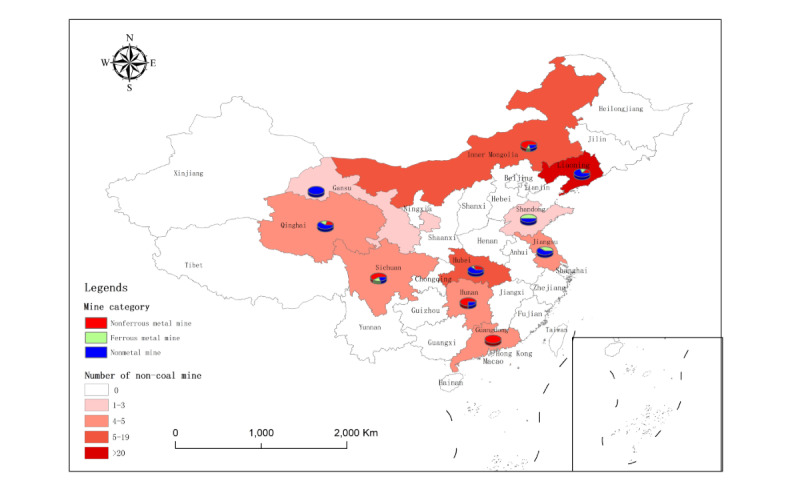
Mine category distribution in the 126 noncoal mines included from 10 provinces.

**Figure 5:**
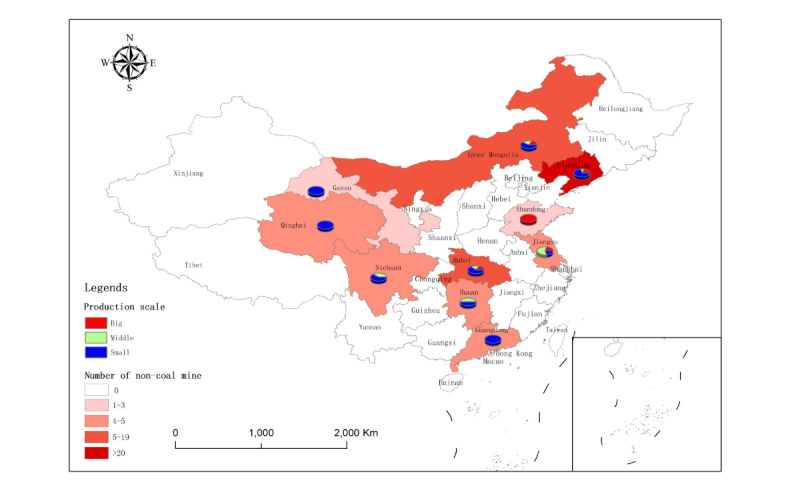
Production scale distribution in the 126 noncoal mines included from 10 provinces.

**Figure 6:**
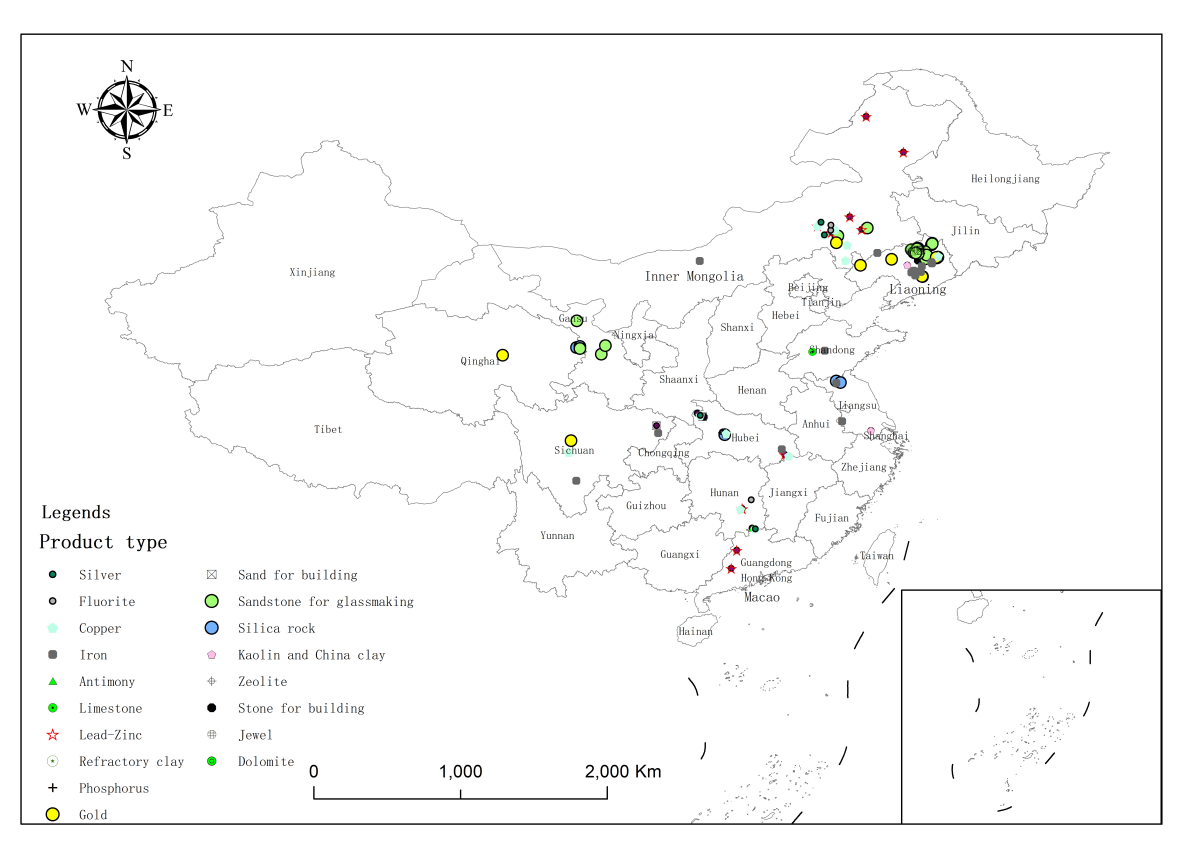
Product type distribution in the 126 noncoal mines included from 10 provinces.

In this cross-sectional study, of the 155 noncoal mines, 22 (14.2%) nonmetal mines and 6 (3.9%) nonferrous metal mines were excluded due to no silica dust exposure (free silica content<10%), and 1 (0.6%) nonferrous metal mine was excluded due to the extremely abnormal concentration of total dust (196.16 mg/m^3^). Ultimately, a total of 126 (81.3%) noncoal mines, with 29,835 miners and 4623 dust samples, were included in this cross-sectional study. Of the 29,835 miners, 13,037 (43.7%) were exposed to silica dust, of whom 12,952 (99.0%) were male. The 126 noncoal mines included were selected from 10 Chinese provinces: (1) 37 (29.4%) nonferrous metal mines from Liaoning, Inner Mongolia, Qinghai, Hubei, Hunan, Sichuan, and Guangdong Provinces; (2) 19 (15.1%) ferrous metal mines from Liaoning, Inner Mongolia, Qinghai, Sichuan, Jiangsu, Hubei, and Shandong Provinces; and (3) 70 (55.6%) nonmetal mines from Liaoning, Inner Mongolia, Qinghai, Gansu, Hubei, Sichuan, Hunan, Jiangsu, and Shandong Provinces. The 4623 dust samples included 1500 (32.4%) dust samples for total dust concentration measurement, 1557 (33.7%) dust samples for respirable dust concentration measurement, and the remaining 1566 (33.9%) dust samples for free silica content determination (see Figures 3-6 and Table S2 in Multimedia Appendix 2).

The overall median PDE, free silica content, total dust concentration, total silica dust concentration, respirable dust concentration, and respirable silica dust concentration were 61.6%, 27.6%, 1.30 mg/m^3^, 0.39 mg/m^3^, 0.58 mg/m^3^, and 0.15 mg/m^3^, respectively. In addition, 963 (64.2%) of total dust samples (concentrations) and 688 (44.2%) of respirable dust samples (concentrations) exceeded China’s OELs, while 1551 (99.6%) of respirable dust samples exceeded the ACGIH REL. Nonmetal mines had the highest median PDE (51.9%), total silica dust concentration (0.48 mg/m^3^), and percentages of total dust samples and respirable dust samples exceeding China’s OELs. Nonferrous metal mines had the highest median total dust concentration (1.61 mg/m^3^) and respirable silica dust concentration (0.17 mg/m^3^). By product type, the highest median concentrations of total dust, total silica dust, respirable dust, and respirable silica dust occurred separately in sand for building (2.85 mg/m^3^), silica rock (1.25 mg/m^3^), zeolite (1.50 mg/m^3^), and silica rock (0.72 mg/m^3^). Small mines had the highest median PDE (64.8%), total dust concentration (1.40 mg/m^3^), total silica dust concentration (0.45 mg/m^3^), respirable silica dust concentration (0.18 mg/m^3^), and (n=621, 71.4%) of total dust samples exceeding China’s OELs. By mining method, open-pit mines had a higher median PDE (66.7%), total silica dust concentration (0.43 mg/m^3^), and percentage (n=597, 65.3%) of total dust samples exceeding China’s OELs; underground mines had higher total dust (1.50 mg/m^3^) and respirable dust (0.66 mg/m^3^) concentrations (see Tables 2 and 3 and Table S3 in Multimedia Appendix 3). By mine category and job, winch control workers of nonmetal mines had the highest total dust concentration (3.13 mg/m^3^) and ratios of total dust concentration (384%) and respirable dust concentration (345.9%) to China’s OELs. Blasters in ferrous metal mines faced the highest respirable dust concentration (2.35 mg/m^3^). Signal workers in both ferrous and nonferrous metal mines had the lowest dust exposure levels (see Figure S1 in Multimedia Appendix 4 and Figure S2 in Multimedia Appendix 5).

**Table 2:** Characteristics of the 126 noncoal mines included.

Characteristics		Mines, n (%)	PDE^a^ (%), median (IQR)
Overall		126 (100.0)	61.6 (49.3-80.0)
**Nonferrous metal mines**		37 (29.4)	60.7 (42.5-73.9)
	Gold	11 (8.7)	60.0 (46.4-66.1)
	Lead-zinc	11 (8.7)	62.9 (59.2-73.9)
	Copper	10 (7.9)	51.9 (31.1-59.3)
	Silver	4 (3.2)	84.7 (63.4-86.1)
	Antimony	1 (0.8)	72.2^b^
Ferrous metal mines (only iron)		19 (15.1)	51.9 (36.0-80.8)
**Nonmetal mines**		70 (55.6)	66.7 (52.9-80.0)
	Stone for building	28 (22.2)	68.3 (53.4-76.0)
	Sandstone for glassmaking	9 (7.1)	80.0 (70.0-82.2)
	Zeolite	5 (4.0)	60.0 (60.0-88.9)
	Silica rock	5 (4.0)	36.6 (26.7-36.7)
	Refractory clay	5 (4.0)	83.3 (66.7-83.3)
	Sand for building	4 (3.2)	70.0 (55.0-81.7)
	Jewels	3 (2.4)	57.5 (41.5-73.4)
	Phosphorus	3 (2.4)	76.9 (67.3-83.4)
	Fluorite	3 (2.4)	66.7 (41.3-83.3)
	Dolomite	2 (1.6)	56.9 (55.6-58.3)
	Kaolin and China clay	2 (1.6)	44.6 (36.3-52.9)
	Limestone	1 (0.8)	57.4^b^
**Production scale**			
	Big	22 (17.5)	57.0 (34.4-79.2)
	Middle	22 (17.5)	63.8 (36.6-78.5)
	Small	82 (65.0)	64.8 (52.9-80.0)
**Mining method**			
	Underground	48 (38.1)	60.4 (37.1-76.5)
	Open pit	78 (61.9)	66.7 (52.7-80.0)

^a^PDE: prevalence of silica dust exposure.

^b^If only 1 mine was included, there was no IQR.

**Table 3:** Dust exposure levels in the 126 noncoal mines included.

Characteristics			Total dust concentration						Respirable dust concentration					
		Dust samples, n (%)		Concentration (mg/m^3^), median (IQR)		Dust samples exceeding China’s OEL^a^, n (%)^b^		Dust samples, n (%)		Concentration (mg/m^3^), median (IQR)		Dust samples exceeding China’s OEL, n (%)^b^		Dust samples exceeding ACGIH^c^ REL^d^, n (%)^b^
Overall			1500 (32.4)		1.30 (0.86-2.48)		963 (64.2)	1557 (33.7)			0.58 (0.33-1.08)		688 (44.2)	1551 (99.6)
**Nonferrous metal mines**			456^e,f^ (9.9)		1.61 (0.86-2.49)		294 (64.5)	498^e,f^ (10.8)			0.68 (0.30-1.19)		249 (50.0)	495 (99.4)
	Gold	123 (2.7)		1.35 (0.92-2.48)		75 (61.0)		123 (2.7)		0.68 (0.61-0.93)		60 (48.8)		123 (100.0)
	Lead-zinc	144^e,f^ (3.1)		1.61 (0.85-2.71)		96 (66.7)		177^f^ (3.8)		0.73 (0.26-1.38)		96 (54.2)		177 (100.0)
	Copper	108^f^ (2.3)		1.77 (0.75-2.37)		72 (66.7)		117 (2.5)		0.63 (0.30-1.35)		57 (48.7)		117 (100.0)
	Silver	45 (1.0)		2.17 (1.15-3.07)		39 (86.7)		45 (1.0)		1.17 (0.82-1.66)		36 (80.0)		45 (100.0)
	Antimony	36 (0.8)		0.78 (0.55-2.18)		12 (33.3)		36 (0.8)		0.22 (0.10-0.38)		0		33 (91.7)
Ferrous metal mines (only iron)			357^e^ (7.7)		1.02 (0.66-2.09)		183 (51.3)	357^e^ (7.7)			0.50 (0.28-0.85)		120 (33.6)	354 (99.2)
**Nonmetal mines**			687^e,f,g^ (14.9)		1.30 (0.92-2.74)		486 (70.7)	702^g^ (15.2)			0.53 (0.37-1.17)		319 (45.4)	702 (100.0)
	Stone for building	285 (6.2)		1.54 (1.16-3.86)		222 (77.9)		285 (6.2)		0.68 (0.37-1.32)		144 (50.5)		285 (100.0)
	Sandstone for glassmaking	105 (2.3)		1.20 (0.88-1.30)		69 (65.7)		105 (2.3)		0.40 (0.37-0.69)		30 (28.6)		105 (100.0)
	Zeolite	45 (1.0)		2.44 (1.26-4.66)		39 (86.7)		45 (1.0)		1.50 (0.43-1.59)		36 (80.0)		45 (100.0)
	Silica rock	45 (1.0)		1.30 (0.38-2.26)		33 (73.3)		45 (1.0)		0.86 (0.44-1.22)		36 (80.0)		45 (100.0)
	Refractory clay	57 (1.2)		1.06 (0.80-1.20)		30 (52.6)		57 (1.2)		0.37 (0.32-0.43)		9 (15.8)		57 (100.0)
	Sand for building	48 (1.0)		2.85 (0.97-4.03)		33 (68.8)		48 (1.0)		0.85 (0.34-1.34)		27 (56.3)		48 (100.0)
	Jewels	18 (0.4)		1.75 (0.99-2.51)		15 (83.3)		18 (0.4)		0.56 (0.49-0.63)		9 (50.0)		18 (100.0)
	Phosphorus	9 (0.2)		0.20 (0.10-2.10)		3 (33.3)		9 (0.2)		0.10 (0.06-1.10)		3 (33.3)		9 (100.0)
	Fluorite	27^f^ (0.6)		0.97 (0.92-3.04)		9 (33.3)		42 (0.9)		0.52 (0.43-1.21)		19 (45.2)		42 (100.0)
	Dolomite	24 (0.5)		1.75 (1.37-2.28)		24 (100.0)		24 (0.5)		0.55 (0.44-0.74)		6 (25.0)		24 (100.0)
	Kaolin and China clay	18 (0.4)		1.00 (0.80-1.31)		9 (50.0)		18 (0.4)		0.55 (0.42-0.59)		0		18 (100.0)
	Limestone	6 (0.1)		0.82 (0.74-0.89)		0		6 (0.1)		0.56 (0.52-0.59)		0		6 (100.0)
**Production scale**														
	Big	363 (7.9)		0.95 (0.61-1.85)		168 (46.3)		363 (7.9)		0.47 (0.27-0.75)		105 (28.9)		360 (99.2)
	Middle	267 (5.8)		1.30 (0.86-2.26)		174 (65.2)		297 (5.8)		0.64 (0.33-1.12)		145 (48.8)		297 (100.0)
	Small	870 (18.8)		1.40 (0.94-2.76)		621 (71.4)		897 (19.4)		0.60 (0.37-1.23)		438 (48.8)		894 (99.7)
**Mining method**														
	Underground	585 (12.7)		1.50 (0.76-2.34)		366 (62.6)		642 (13.9)		0.66 (0.35-1.12)		310 (48.3)		639 (99.5)
	Open pit	915 (19.8)		1.26 (0.86-2.63)		597 (65.3)		915 (19.8)		0.48 (0.33-1.02)		378 (41.3)		912 (99.7)

^a^OEL: occupational exposure limit.

^b^To estimate the overlimit degree of silica dust exposure, we calculated the percentages of dust samples (concentrations) exceeding China’s OEL or exceeding the ACGIH REL.

^c^ACGIH: American Conference for Governmental Industrial Hygienists.

^d^REL: recommended exposure limit.

^e^Some extreme values were excluded.

^f^The total dust concentrations data were lost in 1 copper mine, 1 fluorite mine, and 2 lead-zinc mines.

^g^A total of 9 dust samples were excluded: 3 respirable dust samples’ concentrations were greater than their corresponding total dust concentrations, so the other 3 samples for free silica content determination were also excluded.

### Qualitative and Quantitative Silicosis Risk Assessments of Noncoal Miners

Given that 99.0% of silica dust–exposed miners were male, when predicting the silicosis cumulative HR (H) and incidence (I) over the next 10, 20 and 30 years, we assumed that the gender was male (gender=1). Under total noncoal mine silica dust exposure, the comprehensive risk of silicosis was high (S_weighted_ of ICMM and INDEX assessments was 3.41 and 3.05, respectively), and the cumulative incidence were predicted to be 6.8%, 25.1%, and 49.9% over the next 10, 20, and 30 years, respectively. Under respirable silica dust exposure, the comprehensive risk of silicosis was medium regardless of the mine category, production scale, or mining method, and the cumulative incidence were predicted to be 6.8%, 27.7%, and 57.4% over the next 10, 20, and 30 years, respectively. Nonmetal miners, nonferrous metal miners, small-noncoal-mine workers, and open-pit noncoal miners had a higher risk and cumulative incidence of silicosis. In addition, packing workers and crushers had a higher risk, while signal workers and strokers had a lower risk (see Tables 4-7, Table S4 in Multimedia Appendix 6, and Table S5 in Multimedia Appendix 7).

**Table 4:** Qualitative risk assessment of silicosis under exposure to total noncoal mine silica dust.

Characteristics			ICMM^a^				INDEX^b^		
		Weighted score		Comprehensive risk level		Weighted score		Comprehensive risk level	
Overall			3.41		High		3.05		High
**Mine category**									
	Nonferrous metal	3.56		High		3.37		High	
	Ferrous metal (only iron)	2.96		Medium		2.61		Medium	
	Nonmetal	3.55		High		2.91		Medium	
**Production scale**									
	Big	2.77		Medium		2.54		Medium	
	Middle	3.48		High		3.23		High	
	Small	3.66		High		3.21		High	
**Mining method**									
	Underground	3.46		High		2.86		Medium	
	Open pit	3.38		High		3.17		High	
**Job**									
	Driller	3.70		High		3.12		High	
	Driver	3.21		High		2.90		Medium	
	Blaster	3.00		Medium		2.75		Medium	
	Excavator operator	3.45		High		2.97		Medium	
	Inspector	3.07		High		2.79		Medium	
	Crusher	3.96		High		3.68		High	
	Winch control worker	4.03		Very high		3.48		High	
	Grinder	3.42		High		3.00		Medium	
	Unloader	3.33		High		2.82		Medium	
	Packing worker	4.20		Very high		4.00		High	
	Signal worker	1.75		Low		1.42		Low	
	Screening worker	3.24		High		3.22		High	
	Tailings worker	3.76		High		3.17		High	
	Stroker	1.00		Very low		2.00		Low	

^a^ICMM: International Mining and Metals Commission’s risk-rating table.

^b^INDEX: occupational hazard risk index.

**Table 5:** Quantitative risk assessment of silicosis under exposure to total noncoal mine silica dust.

Characteristics		Cumulative HR^a^ (%), median (IQR)			Cumulative incidence (%), median (IQR)		
		10 years	20 years	30 years	10 years	20 years	30 years
Overall		0.07 (0.06-0.09)	0.30 (0.22-0.47)	0.69 (0.45-1.42)	6.8 (5.9-8.5)	25.1 (19.6-37.3)	49.9 (36.3-75.8)
**Mine category**							
	Nonferrous metal	0.07 (0.06-0.09)	0.28 (0.20-0.46)	0.66 (0.40-1.37)	6.7(5.7-8.5)	24.4 (18.3-36.7)	48.3 (33.2-74.8)
	Ferrous metal (only iron)	0.06 (0.06-0.07)	0.24 (0.21-0.31)	0.51 (0.41-0.78)	6.1 (5.7-7)	20.9 (18.5-26.8)	39.7 (33.9-54)
	Nonmetal	0.07 (0.07-0.10)	0.33 (0.26-0.63)	0.84 (0.59-2.24)	7.2 (6.5-9.9)	28.0 (23-46.9)	56.8 (44.8-89.4)
**Production scale**							
	Big	0.06 (0.06-0.07)	0.23 (0.20-0.32)	0.50 (0.40-0.80)	6.1 (5.7-7.1)	20.8 (18.2-27.2)	39.4 (33.1-54.9)
	Middle	0.07 (0.06-0.09)	0.28 (0.23-0.51)	0.66 (0.48-1.61)	6.7 (6.1-8.9)	24.4 (20.4-39.8)	48.1 (38.4-80.1)
	Small	0.07 (0.06-0.10)	0.32 (0.24-0.58)	0.79 (0.51-1.98)	7.1 (6.2-9.5)	27.0 (21.1-44.2)	54.5 (40.0-86.2)
**Mining method**							
	Underground	0.07 (0.06-0.08)	0.26 (0.21-0.37)	0.60 (0.41-0.99)	6.5 (5.8-7.6)	23.2 (18.6-30.7)	45.4 (34-62.8)
	Open pit	0.07 (0.06-0.10)	0.31 (0.24-0.56)	0.76 (0.51-1.88)	7.0 (6.1-9.3)	26.5 (20.9-43.1)	53.3 (39.7-84.8)
**Job**							
	Driller	0.07 (0.06-0.10)	0.31 (0.25-0.57)	0.75 (0.55-1.93)	7.0 (6.3-9.4)	26.3 (21.9-43.6)	52.8 (42.2-85.5)
	Driver	0.07 (0.06-0.08)	0.29 (0.23-0.34)	0.69 (0.48-0.90)	6.8 (6.1-7.4)	25.1 (20.4-29.1)	49.9 (38.4-59.2)
	Blaster	0.07 (0.06-0.08)	0.27 (0.21-0.36)	0.63 (0.42-0.94)	6.6 (5.8-7.5)	23.8 (18.7-30)	46.7 (34.3-61.1)
	Excavator operator	0.07 (0.06-0.08)	0.27 (0.21-0.34)	0.61 (0.43-0.86)	6.5 (5.8-7.3)	23.3 (19-28.5)	45.6 (35-57.9)
	Inspector	0.07 (0.06-0.08)	0.25 (0.2-0.4)	0.56 (0.40-1.11)	6.3 (5.7-7.9)	22.2 (18.1-32.7)	42.8 (32.9-67)
	Crusher	0.09 (0.07-0.13)	0.43 (0.27-0.98)	1.24 (0.60-4.31)	8.2 (6.5-12.1)	34.8 (23.2-62.4)	71.2 (45.4-98.7)
	Winch control worker	0.07 (0.06-0.86)	0.32 (0.24-43.61)	0.81 (0.53-1275.76)	7.1 (6.2-57.8)	27.5 (21.5-100)	55.7 (41.2-100)
	Grinder	0.07 (0.06-0.09)	0.28 (0.2-0.51)	0.65 (0.39-1.60)	6.7 (5.6-8.9)	24.3 (17.8-39.7)	48.1 (32-79.9)
	Unloader	0.07 (0.06-0.09)	0.28 (0.21-0.43)	0.67 (0.43-1.25)	6.7 (5.8-8.2)	24.7 (19.1-35)	49 (35.2-71.5)
	Packing worker	0.08 (0.08-0.16)	0.38 (0.37-1.43)	1.02 (0.98-7.58)	7.7 (7.6-14.5)	31.3 (30.5-76)	64.1 (62.4-99.9)
	Signal worker	0.06 (0.05-0.06)	0.20 (0.18-0.21)	0.40 (0.33-0.44)	5.7 (5.3-5.9)	18.3 (16-19.2)	33.2 (27.8-35.6)
	Screening worker	0.07 (0.06-0.13)	0.29 (0.20-0.98)	0.68 (0.39-4.30)	6.7 (5.7-12.1)	24.8 (18-62.4)	49.2 (32.5-98.6)
	Tailings worker	0.08 (0.06-0.11)	0.39 (0.20-0.75)	1.10 (0.38-2.86)	7.8 (5.6-10.7)	32.2 (17.7-52.5)	65.3 (31.8-94.3)
	Stroker	0.06^b^	0.19^b^	0.38^b^	5.6^b^	17.6^b^	31.5^b^

^a^HR: hazard ratio.

^b^If only 1 job was included, there was no IQR.

**Table 6:** Qualitative risk assessment of silicosis under exposure to respirable noncoal mine silica dust.

Characteristics			ICMM^a^				INDEX^b^		
		Weighted score		Comprehensive risk level		Weighted score		Comprehensive risk level	
Overall			2.62		Medium		2.55		Medium
**Mine category**									
	Nonferrous metal	2.65		Medium		2.82		Medium	
	Ferrous metal (only iron)	2.18		Medium		2.06		Medium	
	Nonmetal	2.91		Medium		2.51		Medium	
**Production scale**									
	Big	2.09		Medium		2.03		Medium	
	Middle	2.77		Medium		2.74		Medium	
	Small	2.79		Medium		2.69		Medium	
**Mining method**									
	Underground	2.82		Medium		2.47		Medium	
	Open pit	2.49		Medium		2.60		Medium	
**Job**									
	Driller	3.45		High		2.70		Medium	
	Driver	2.00		Low		2.37		Medium	
	Blaster	2.63		Medium		2.75		Medium	
	Excavator operator	2.00		Low		2.45		Medium	
	Inspector	2.40		Medium		2.16		Medium	
	Crusher	3.73		High		3.05		High	
	Winch control worker	3.67		High		3.36		High	
	Grinder	2.61		Medium		2.53		Medium	
	Unloader	2.52		Medium		2.36		Medium	
	Packing worker	4.20		very high		3.87		High	
	Signal worker	1.50		Low		1.25		Low	
	Screening worker	2.89		Medium		2.72		Medium	
	Tailings worker	3.29		High		2.83		Medium	
	Stroker	1.00		Very low		1.67		Low	

^a^ICMM: International Mining and Metals Commission’s risk-rating table.

^b^INDEX: occupational hazard risk index.

**Table 7:** Quantitative risk assessment of silicosis under exposure to respirable noncoal mine silica dust.

Characteristics		Cumulative HR^a^ (%), median (IQR)			Cumulative incidence (%), median (IQR)		
		10 years	20 years	30 years	10 years	20 years	30 years
Overall		0.07 (0.06-0.10)	0.32 (0.24-0.70)	0.85 (0.54-2.69)	6.8 (5.9-9.9)	27.7 (21.2-50.2)	57.4 (41.6-93.2)
**Mine category**							
	Nonferrous metal	0.07 (0.06-0.11)	0.35 (0.21-0.71)	0.94 (0.44-2.77)	7.1 (5.5-10)	29.3 (18.8-51)	60.9 (35.5-93.8)
	Ferrous metal (only iron)	0.06 (0.06-0.08)	0.26 (0.21-0.39)	0.62 (0.45-1.12)	6.2 (5.6-7.5)	23.2 (19-32.3)	46.5 (36.1-67.5)
	Nonmetal	0.07 (0.07-0.12)	0.34 (0.27-0.94)	0.94 (0.67-4.21)	7.1 (6.3-11.4)	29.2 (24.0-61.0)	60.8 (48.6-98.5)
**Production scale**							
	Big	0.06 (0.06-0.07)	0.26 (0.20-0.35)	0.62 (0.43-0.96)	6.2 (5.5-7.1)	23.1 (18.5-29.6)	46.3 (34.8-61.8)
	Middle	0.07 (0.06-0.11)	0.34 (0.25-0.71)	0.90 (0.57-2.77)	7.0 (6.0-10.0)	28.6 (21.9-50.9)	59.5 (43.3-93.8)
	Small	0.07 (0.06-0.11)	0.36 (0.26-0.93)	0.98 (0.60-4.17)	7.2 (6.1-11.3)	30.0 (22.7-60.7)	62.5 (45.2-98.5)
**Mining method**							
	Underground	0.07 (0.06-0.10)	0.32 (0.22-0.59)	0.83 (0.47-2.12)	6.8 (5.7-9.2)	27.3 (19.6-44.9)	56.4 (37.5-88)
	Open pit	0.07 (0.06-0.11)	0.33 (0.26-0.79)	0.86 (0.60-3.22)	6.9 (6.1-10.5)	27.8 (22.6-54.4)	57.5 (44.9-96)
**Job**							
	Driller	0.09 (0.07-0.15)	0.51 (0.28-1.39)	1.69 (0.67-7.54)	8.5 (6.3-13.6)	40.1 (24.1-75)	81.6 (48.8-99.9)
	Driver	0.07 (0.06-0.08)	0.30 (0.25-0.38)	0.78 (0.57-1.07)	6.60 (6.0-7.4)	26.3 (22.0-31.5)	54.0 (43.6-65.8)
	Blaster	0.09 (0.06-0.12)	0.47 (0.24-0.92)	1.51 (0.56-4.11)	8.20 (6.0-11.3)	37.3 (21.7-60.4)	75.8 (42.7-98.4)
	Excavator operator	0.07 (0.06-0.09)	0.28 (0.22-0.48)	0.68 (0.48-1.53)	6.4 (5.7-8.3)	24.4 (19.9-38)	49.4 (38.3-78.3)
	Inspector	0.07 (0.06-0.09)	0.28 (0.23-0.49)	0.69 (0.49-1.61)	6.4 (5.7-8.4)	24.5 (20.2-39)	49.6 (39-79.9)
	Crusher	0.11 (0.06-0.16)	0.71 (0.26-1.55)	2.76 (0.61-8.91)	10 (6.1-14.4)	50.9 (22.8-78.8)	93.7 (45.4-100.0)
	Winch control worker	0.09 (0.07-1.06)	0.51 (0.34-71.92)	1.68 (0.92-2819.41)	8.5 (7.0-65.2)	39.9 (28.9-100.0)	81.3 (60.1-100.0)
	Grinder	0.07 (0.05-0.12)	0.35 (0.19-0.94)	0.95 (0.38-4.23)	7.1 (5.3-11.4)	29.4 (17.1-61.1)	61.3 (31.4-98.5)
	Unloader	0.08 (0.06-0.09)	0.37 (0.24-0.56)	1.05 (0.53-1.95)	7.3 (5.9-8.9)	31.2 (21.1-43.1)	65.1 (41.2-85.8)
	Packing worker	0.11 (0.11-0.2)	0.84 (0.79-2.58)	3.58 (3.22-19.16)	10.8 (10.5-18.1)	57.0 (54.5-92.5)	97.2 (96-100)
	Signal worker	0.06 (0.05-0.06)	0.20 (0.16-0.25)	0.41 (0.31-0.57)	5.4 (4.9-6.0)	17.9 (15.2-21.8)	33.3 (26.5-43.0)
	Screening worker	0.07 (0.06-0.13)	0.32 (0.21-1.06)	0.86 (0.45-5.07)	6.90 (5.6-12.1)	27.8 (19.0-65.5)	57.5 (35.9-99.4)
	Tailings worker	0.09 (0.06-0.13)	0.52 (0.2-1.04)	1.73 (0.42-4.88)	8.60 (5.5-11.9)	40.5 (18.4-64.6)	82.2 (34.5-99.2)
	Stroker	0.05^b^	0.19^b^	0.38^b^	5.3^b^	17.2^b^	31.5^b^

^a^HR: hazard ratio.

^b^If only 1 job was included, there was no IQR.

## Discussion

### Principal Findings

By using nonprobability convenience-based sampling, 155 noncoal mines were selected from 10 provinces in northern, western, southern, eastern, and central China, and 126 noncoal mines were included in this study. We found that Chinese noncoal miners still suffer high-level silica dust exposure and medium-level silicosis risk, especially miners in nonmetal, nonferrous metal, small, and open-pit noncoal mines. In addition, the silicosis cumulative incidence caused by total silica dust exposure is predicted to be 6.8%, 25.1%, and 49.9% over the next 10, 20, and 30 years, respectively, and the cumulative incidences caused by respirable silica dust exposure is predicted to be 6.8%, 27.7%, and 57.4%, respectively. These findings are of great reference value for improving occupational health in China, especially for implementing the Occupational Health Protection Action (2019-2030).

### Comparison With Prior Work

Silica is the key component of dust in a mine working environment. Silicosis, a preventable lung disease, has been a serious public health issue worldwide for many decades. The recent outbreaks of silicosis have made governments and scientists frustrated and confused [1,14,40], and high incidences of silicosis in Asia, Africa, and South America are particularly concerning [40]. Occupational health plays an important role in China’s broader health strategy. Assessment of occupational health risk is deemed 1 of the tasks of the health administrative department. Therefore, it is vital to conduct surveillance of the risk of silicosis in Chinese noncoal mines. Many previous medical studies have focused on the effect of noncoal mine silica dust exposure on silicosis morbidity and mortality; however, the visibility of silicosis risk has never been complete or consistent. This study established prediction formulas of quantitative silicosis risk, distinguishing gender and silica dust types. Kaplan-Meier survival analysis showed that male noncoal miners have a higher silicosis probability than female noncoal miners. The finding is consistent with previously published results that men have a 77% higher incidence of silicosis than women [41]. However, we did not find significant difference among different mine categories or smoking histories. This result might possibly be explained by the healthy worker survivor effect. In this cross-sectional study, most silica dust–exposed miners were male. This is consistent with the reality in China. Therefore, when predicting the silicosis cumulative HR and incidence over the next 10, 20, and 30 years, we assumed that the gender was male.

A previous study based on 29 Chinese metal mines and pottery factories indicated that the mean respirable silica dust concentration of the mines fell to less than 0.1 mg/m^3^ after 1970 and that the concentrations of pottery factories ranged from 0.12 to 0.15 mg/m^3^ after 1990 [22]. The updated study, based on 1 iron mine, also showed that the mean respirable silica dust concentration gradually decreased to below 0.01 mg/m^3^ in 2012 [42]. However, survey data of our cross-sectional study showed that 64.2% of total dust samples and 44.2% of respirable dust samples exceeded China’s OELs and that 99.6% of respirable dust samples exceeded the ACGIH REL. In addition, workers in nonmetal, nonferrous metal, small noncoal mines, and open-pit noncoal mines suffered high silica dust exposure levels. This was consistent with results of qualitative and quantitative silicosis risk assessments. The most probable reason for the different findings is the differences in mining technology levels, which would normally be related to the regional economic development in China. Moreover, our survey data were collected from northern, western, southern, eastern, and central China, including nonferrous metal, ferrous metal, and nonmetal mines, not just central and southern China.

It should be noted that the latest WHO/ILO joint estimates indicated that the pooled prevalence of occupational exposure to silica dust in mining was 0.75 and that the pooled level of silica dust was 0.04 mg/m^3^ [42]. The quality of the prevalence evidence is moderate. More worryingly, the quality of the level evidence is low. Our study showed that Chinese noncoal mines have lower prevalence (61.6%) and higher levels of occupational exposure to silica dust. Furthermore, the overall total dust concentration was 1.30 mg/m^3^, and the respirable dust concentration was 0.58 mg/m^3^. It is foreseeable that these findings would be high-quality evidence for both prevalence and level estimation of exposure to silica dust in noncoal mines.

### Limitations

Although this study provides novel findings on the risk of silicosis in Chinese noncoal miners, several limitations need to be considered regarding these findings. The estimation formula of the historical respirable concentration (before 1992) was rough without distinguishing the mining method, mine category, or production scale. Furthermore, only 1 antimony mine and 1 limestone mine were included, limiting the representativeness of these 2 types of mines. Finally, compared to the United States, the accuracy of Chinese sampling procedures might be lower.

### Conclusion

An interesting result was observed in the qualitative risk assessment. The qualitative risks of silicosis caused by total silica dust exposure and respirable silica dust exposure were high and medium, respectively. This result demonstrates that both total silica dust and respirable silica dust data are vital for occupational health risk assessment, rather than respirable silica dust data alone. Quantitative risk assessment showed that the silicosis cumulative incidence caused by total silica dust exposure and respirable silica dust exposure would be up to 49.9% and 57.4% over the next 30 years. These findings suggest that silica dust exposure and silicosis risk in Chinese noncoal mines remain a problem. As China has yet to completely fulfill the National Occupational Disease Prevention and Control Plan (2021-2025), these nationally representative data of noncoal mine silica dust exposure would provide valuable evidence for improving miners’ working environments and reducing the risk of silicosis. Effective control measures for reducing noncoal mine silica dust levels, especially primary prevention strategies, are critical to achieve the goal of improving occupational health in China.

## Appendix

Multimedia Appendix 1 Location, mining method, product type, mine category, and production scale of 3 noncoal mines in which the retrospective cohort study was conducted.

Multimedia Appendix 2 Location, mining method, product type, mine category, production scale, and the number of workers in the included 126 noncoal mines in which the cross-sectional study was conducted.

Multimedia Appendix 3 Number of workers and dust exposure levels in the 126 noncoal mines included.

Multimedia Appendix 4 Concentration of total dust and ratio of total dust concentration to China’s occupational exposure limit (OEL) by mine category and job.

Multimedia Appendix 5 Concentration of respirable dust and ratio of respirable dust concentration to China’s occupational exposure limit (OEL) by mine category and job.

Multimedia Appendix 6 Under exposure to total noncoal mine silica dust, the raw 5-level risk assessment results of the ICMM and INDEX. ICMM: International Mining and Metals Commission's risk-rating table; INDEX: occupational hazard risk index.

Multimedia Appendix 7 Under exposure to respirable noncoal mine silica dust, the raw 5-level risk assessment results of the ICMM and INDEX. ICMM: International Mining and Metals Commission's risk-rating table; INDEX: occupational hazard risk index.

## Abbreviations

ACGIH: American Conference for Governmental Industrial Hygienists
HR: hazard ratio
ICMM: International Mining and Metals Commission’s risk-rating table
ILO: International Labour Organization
INDEX: occupational hazard risk index
JEM: job exposure matrix
OEL: occupational exposure limit
PDE: prevalence of silica dust exposure
REL: recommended exposure limit
WHO: World Health Organization

## Data Availability

The data sets generated and analyzed during this study are available from the corresponding author upon reasonable request.
