# Supplementary material for: Occupational Exposure to Silica Dust and Silicosis Risk in Chinese Noncoal Mines: Qualitative and Quantitative Risk Assessment
Source: JMIR Public Health Surveill. 2024 Sep 2;10:e56283. doi: 10.2196/56283 (PMC11406111; doi:10.2196/56283)
Supplement: Multimedia Appendix 1 [file publichealth_v10i1e56283_app1.doc]

Table S1. Location, mining method, product type, mine category, and production scale of three non-coal mines which the retrospective cohort study was conducted in.

| Non-coal mine number | Province | City | Mining method | Product type | Mine category | Production scale |
| --- | --- | --- | --- | --- | --- | --- |
| 1 | Hubei | Huangshi | Underground | Copper | Nonferrous metal mine | Middle |
| 2 | Hubei | Huangshi | Underground and open-pit | Copper and iron | Nonferrous metal mine and ferrous metal mine | Big |
| 3 | Hubei | Huangshi | Underground and open-pit | Copper | Nonferrous metal mine | Middle |
