# Supplementary material for: Occupational Exposure to Silica Dust and Silicosis Risk in Chinese Noncoal Mines: Qualitative and Quantitative Risk Assessment
Source: JMIR Public Health Surveill. 2024 Sep 2;10:e56283. doi: 10.2196/56283 (PMC11406111; doi:10.2196/56283)
Supplement: Multimedia Appendix 2 [file publichealth_v10i1e56283_app2.doc]

**Table S2. Location, mining method, product type, mine category, production scale, and the number of workers of the included 126 non-coal mines which the cross-sectional study was conducted in. No: Number.**

| Non-coal mine number | Province | City | Mining method | Product type | Mine category | Production scale | No of workers | No of female workers |
| --- | --- | --- | --- | --- | --- | --- | --- | --- |
| 1 | Liaoning | Fushun | Underground | Gold | Nonferrous metal mine | Small | 53 | 6 |
| 2 | Liaoning | Fushun | Open-pit | Gold | Nonferrous metal mine | Small | 31 | 0 |
| 3 | Liaoning | Fushun | Underground | Copper | Nonferrous metal mine | Small | 81 | 0 |
| 4 | Sichuan | Aba (Ngawa) Tibetan and Qiang Autonomous Prefecture | Underground | Gold | Nonferrous metal mine | Small | 34 | 4 |
| 5 | Sichuan | Liangshan Yi Autonomous Prefecture | Underground | Iron | Ferrous metal mine | Small | 52 | 4 |
| 6 | Sichuan | Bazhong | Open-pit | Sand for building | Nonmetal mine | Small | 12 | 1 |
| 7 | Liaoning | Anshan | Open-pit | Iron | Ferrous metal mine | Big | 1151 | 0 |
| 8 | Liaoning | Anshan | Open-pit | Iron | Ferrous metal mine | Big | 1031 | 67 |
| 9 | Liaoning | Anshan | Open-pit | Iron | Ferrous metal mine | Big | 718 | 49 |
| 10 | Liaoning | Tieling | Open-pit | Stone for building | Nonmetal mine | Small | 14 | 0 |
| 11 | Liaoning | Tieling | Open-pit | Stone for building | Nonmetal mine | Small | 7 | 0 |
| 12 | Liaoning | Tieling | Open-pit | Stone for building | Nonmetal mine | Small | 5 | 0 |
| 13 | Liaoning | Tieling | Open-pit | Stone for building | Nonmetal mine | Small | 20 | 0 |
| 14 | Liaoning | Fushun | Open-pit | Stone for building | Nonmetal mine | Small | 12 | 0 |
| 15 | Liaoning | Fushun | Open-pit | Dolomite | Nonmetal mine | Small | 9 | 0 |
| 16 | Liaoning | Fushun | Open-pit | Dolomite | Nonmetal mine | Small | 12 | 0 |
| 17 | Liaoning | Tieling | Open-pit | Stone for building | Nonmetal mine | Small | 10 | 0 |
| 18 | Liaoning | Tieling | Open-pit | Stone for building | Nonmetal mine | Middle | 7 | 0 |
| 19 | Hunan | Hengyang | Underground | Lead-zinc | Nonferrous metal mine | Small | 160 | 56 |
| 20 | Hunan | Hengyang | Underground | Lead-zinc | Nonferrous metal mine | Middle | 350 | 60 |
| 21 | Hunan | Hengyang | Underground | Copper | Nonferrous metal mine | Small | 280 | 50 |
| 22 | Hunan | Hengyang | Underground | Fluorite | Nonmetal mine | Middle | 350 | 75 |
| 23 | Liaoning | Shenyang | Open-pit | Sand for building | Nonmetal mine | Small | 10 | 0 |
| 24 | Liaoning | Shenyang | Open-pit | Stone for building | Nonmetal mine | Small | 12 | 0 |
| 25 | Liaoning | Shenyang | Open-pit | Zeolite | Nonmetal mine | Small | 10 | 0 |
| 26 | Liaoning | Shenyang | Open-pit | Sandstone for glassmaking | Nonmetal mine | Small | 8 | 0 |
| 27 | Liaoning | Shenyang | Open-pit | Zeolite | Nonmetal mine | Small | 9 | 4 |
| 28 | Liaoning | Shenyang | Open-pit | Zeolite | Nonmetal mine | Small | 9 | 0 |
| 29 | Liaoning | Shenyang | Open-pit | Zeolite | Nonmetal mine | Small | 5 | 0 |
| 30 | Liaoning | Shenyang | Open-pit | Stone for building | Nonmetal mine | Small | 5 | 0 |
| 31 | Liaoning | Shenyang | Open-pit | Stone for building | Nonmetal mine | Big | 20 | 0 |
| 32 | Liaoning | Shenyang | Open-pit | Stone for building | Nonmetal mine | Big | 13 | 0 |
| 33 | Liaoning | Shenyang | Open-pit | Stone for building | Nonmetal mine | Small | 9 | 0 |
| 34 | Liaoning | Shenyang | Open-pit | Zeolite | Nonmetal mine | Small | 5 | 0 |
| 35 | Liaoning | Shenyang | Open-pit | Refractory clay | Nonmetal mine | Middle | 12 | 0 |
| 36 | Liaoning | Shenyang | Open-pit | Refractory clay | Nonmetal mine | Middle | 12 | 0 |
| 37 | Liaoning | Shenyang | Open-pit | Sandstone for glassmaking | Nonmetal mine | Small | 13 | 4 |
| 38 | Liaoning | Shenyang | Open-pit | Sandstone for glassmaking | Nonmetal mine | Small | 10 | 4 |
| 39 | Liaoning | Shenyang | Open-pit | Sandstone for glassmaking | Nonmetal mine | Small | 35 | 0 |
| 40 | Liaoning | Shenyang | Open-pit | Refractory clay | Nonmetal mine | Small | 12 | 0 |
| 41 | Liaoning | Shenyang | Open-pit | Refractory clay | Nonmetal mine | Small | 15 | 0 |
| 42 | Liaoning | Shenyang | Open-pit | Sandstone for glassmaking | Nonmetal mine | Small | 15 | 0 |
| 43 | Liaoning | Shenyang | Open-pit | Stone for building | Nonmetal mine | Small | 15 | 0 |
| 44 | Liaoning | Shenyang | Open-pit | Stone for building | Nonmetal mine | Small | 12 | 0 |
| 45 | Liaoning | Shenyang | Open-pit | Stone for building | Nonmetal mine | Small | 17 | 0 |
| 46 | Liaoning | Shenyang | Open-pit | Sandstone for glassmaking | Nonmetal mine | Small | 40 | 0 |
| 47 | Liaoning | Shenyang | Open-pit | Stone for building | Nonmetal mine | Small | 17 | 0 |
| 48 | Liaoning | Shenyang | Open-pit | Stone for building | Nonmetal mine | Small | 26 | 0 |
| 49 | Liaoning | Shenyang | Open-pit | Sandstone for glassmaking | Nonmetal mine | Small | 45 | 15 |
| 50 | Liaoning | Shenyang | Open-pit | Stone for building | Nonmetal mine | Small | 8 | 4 |
| 51 | Liaoning | Shenyang | Open-pit | Stone for building | Nonmetal mine | Small | 12 | 0 |
| 52 | Liaoning | Shenyang | Open-pit | Refractory clay | Nonmetal mine | Small | 15 | 0 |
| 53 | Liaoning | Shenyang | Open-pit | Sandstone for glassmaking | Nonmetal mine | Small | 12 | 0 |
| 54 | Liaoning | Shenyang | Open-pit | Sandstone for glassmaking | Nonmetal mine | Small | 17 | 0 |
| 55 | Liaoning | Shenyang | Open-pit | Stone for building | Nonmetal mine | Small | 11 | 0 |
| 56 | Liaoning | Shenyang | Open-pit | Kaolin and China clay | Nonmetal mine | Big | 17 | 0 |
| 57 | Liaoning | Shenyang | Open-pit | Stone for building | Nonmetal mine | Small | 4 | 0 |
| 58 | Liaoning | Shenyang | Open-pit | Stone for building | Nonmetal mine | Small | 4 | 0 |
| 59 | Liaoning | Fushun | Underground | Gold | Nonferrous metal mine | Big | 87 | 0 |
| 60 | Liaoning | Fushun | Open-pit | Iron | Ferrous metal mine | Middle | 343 | 0 |
| 61 | Liaoning | Fushun | Underground | Iron | Ferrous metal mine | Middle | 343 | 0 |
| 62 | Liaoning | Chaoyang | Underground | Gold | Nonferrous metal mine | Small | 53 | 3 |
| 63 | Liaoning | Fushun | Underground | Iron | Ferrous metal mine | Small | 42 | 3 |
| 64 | Liaoning | Liaoyang | Open-pit | Iron | Ferrous metal mine | Middle | 180 | 11 |
| 65 | Liaoning | Tieling | Open-pit | Sand for building | Nonmetal mine | Small | 18 | 0 |
| 66 | Liaoning | Fuxin | Underground | Gold | Nonferrous metal mine | Middle | 104 | 9 |
| 67 | Liaoning | Liaoyang | Underground | Iron | Ferrous metal mine | Small | 49 | 0 |
| 68 | Liaoning | Liaoyang | Underground | Iron | Ferrous metal mine | Big | 1649 | 78 |
| 69 | Liaoning | Liaoyang | Open-pit | Iron | Ferrous metal mine | Big | 1493 | 70 |
| 70 | Liaoning | Dandong | Underground | Gold | Nonferrous metal mine | Big | 133 | 3 |
| 71 | Liaoning | Dandong | Underground | Gold | Nonferrous metal mine | Big | 137 | 0 |
| 72 | Liaoning | Anshan | Open-pit | Iron | Ferrous metal mine | Big | 1717 | 202 |
| 73 | Liaoning | Chaoyang | Underground | Gold | Nonferrous metal mine | Small | 20 | 1 |
| 74 | Jiangsu | Nanjing | Open-pit | Kaolin and China clay | Nonmetal mine | Middle | 1200 | 36 |
| 75 | Jiangsu | Nanjing | Underground | Iron | Ferrous metal mine | Big | 3600 | 216 |
| 76 | Jiangsu | Xinyi | Open-pit | Silica rock | Nonmetal mine | Middle | 80 | 5 |
| 77 | Jiangsu | Xinyi | Open-pit | Silica rock | Nonmetal mine | Middle | 676 | 40 |
| 78 | Jiangsu | Xinyi | Open-pit | Iron | Ferrous metal mine | Small | 50 | 0 |
| 79 | Inner Mongolia | Baotou | Underground | Iron | Ferrous metal mine | Big | 3695 | 221 |
| 80 | Inner Mongolia | Chifeng | Underground | Iron | Ferrous metal mine | Small | 33 | 0 |
| 81 | Inner Mongolia | Chifeng | Underground | Copper | Nonferrous metal mine | Big | 545 | 16 |
| 82 | Inner Mongolia | Chifeng | Underground | Lead-zinc | Nonferrous metal mine | Small | 56 | 0 |
| 83 | Inner Mongolia | Chifeng | Open-pit | Stone for building | Nonmetal mine | Small | 6 | 0 |
| 84 | Inner Mongolia | Chifeng | Underground | Lead-zinc | Nonferrous metal mine | Middle | 722 | 43 |
| 85 | Inner Mongolia | Chifeng | Open-pit | Fluorite | Nonmetal mine | Small | 36 | 0 |
| 86 | Inner Mongolia | Chifeng | Open-pit | Stone for building | Nonmetal mine | Small | 23 | 0 |
| 87 | Inner Mongolia | Chifeng | Underground | Lead-zinc | Nonferrous metal mine | Small | 116 | 0 |
| 88 | Inner Mongolia | Chifeng | Underground | Copper | Nonferrous metal mine | Big | 1400 | 84 |
| 89 | Inner Mongolia | Chifeng | Open-pit | Fluorite | Nonmetal mine | Small | 21 | 0 |
| 90 | Inner Mongolia | Chifeng | Underground | Copper | Nonferrous metal mine | Small | 133 | 0 |
| 91 | Inner Mongolia | Chifeng | Underground | Silver | Nonferrous metal mine | Middle | 368 | 0 |
| 92 | Inner Mongolia | Chifeng | Underground | Silver | Nonferrous metal mine | Middle | 362 | 0 |
| 93 | Inner Mongolia | Chifeng | Underground | Lead-zinc | Nonferrous metal mine | Small | 97 | 0 |
| 94 | Inner Mongolia | Chifeng | Underground | Gold | Nonferrous metal mine | Middle | 247 | 0 |
| 95 | Inner Mongolia | Chifeng | Underground | Copper | Nonferrous metal mine | Small | 19 | 0 |
| 96 | Inner Mongolia | Hulun Buir | Underground | Lead-zinc | Nonferrous metal mine | Middle | 420 | 0 |
| 97 | Inner Mongolia | Hulun Buir | Underground | Lead-zinc | Nonferrous metal mine | Small | 47 | 0 |
| 98 | Qinghai | Haidong | Open-pit | Silica rock | Nonmetal mine | Small | 41 | 0 |
| 99 | Qinghai | Haidong | Open-pit | Silica rock | Nonmetal mine | Small | 15 | 0 |
| 100 | Qinghai | Haidong | Open-pit | Stone for building | Nonmetal mine | Small | 21 | 0 |
| 101 | Qinghai | Haixi Mongolian and Tibetan Autonomous Prefecture | Underground | Gold | Nonferrous metal mine | Small | 196 | 0 |
| 102 | Qinghai | Haixi Mongolian and Tibetan Autonomous Prefecture | Underground | Iron | Ferrous metal mine | Small | 57 | 0 |
| 103 | Sichuan | Kangding | Underground | Copper | Nonferrous metal mine | Middle | 431 | 143 |
| 104 | Sichuan | Ganzi (Garzê) Tibetan Autonomous Prefecture | Underground | Copper | Nonferrous metal mine | Middle | 330 | 91 |
| 105 | Hubei | Shiyan | Underground | Silver | Nonferrous metal mine | Middle | 300 | 8 |
| 106 | Hubei | Shiyan | Open-pit | Jewel | Nonmetal mine | Small | 196 | 5 |
| 107 | Hubei | Shiyan | Open-pit | Jewel | Nonmetal mine | Small | 40 | 2 |
| 108 | Hubei | Shiyan | Open-pit | Jewel | Nonmetal mine | Small | 28 | 4 |
| 109 | Hubei | Shiyan | Open-pit | Sand for building | Nonmetal mine | Big | 25 | 0 |
| 110 | Hubei | Huangshi | Underground | Copper | Nonferrous metal mine | Big | 848 | 0 |
| 111 | Hubei | Yichang | Open-pit | Silica rock | Nonmetal mine | Small | 15 | 0 |
| 112 | Hubei | Ezhou | Underground | Iron | Ferrous metal mine | Middle | 130 | 0 |
| 113 | Hubei | Huangshi | Underground | Lead-zinc | Nonferrous metal mine | Small | 98 | 0 |
| 114 | Hubei | Yichang | Underground | Copper | Nonferrous metal mine | Small | 55 | 6 |
| 115 | Hubei | Yichang | Open-pit | Phosphorus | Nonmetal mine | Small | 200 | 24 |
| 116 | Hubei | Yichang | Open-pit | Phosphorus | Nonmetal mine | Small | 45 | 9 |
| 117 | Hubei | Yichang | Open-pit | Phosphorus | Nonmetal mine | Middle | 251 | 2 |
| 118 | Guangdong | Yunfu | Underground | Lead-zinc | Nonferrous metal mine | Small | 263 | 0 |
| 119 | Guangdong | Zhaoqing | Underground | Lead-zinc | Nonferrous metal mine | Small | 20 | 1 |
| 120 | Guangdong | Lechang | Underground | Silver | Nonferrous metal mine | Small | 21 | 0 |
| 121 | Guangdong | Lechang | Underground | Antimony | Nonferrous metal mine | Small | 18 | 0 |
| 122 | Gansu | Lanzhou | Open-pit | Stone for building | Nonmetal mine | Small | 9 | 0 |
| 123 | Gansu | Baiyin | Open-pit | Stone for building | Nonmetal mine | Small | 14 | 0 |
| 124 | Gansu | Wuwei | Open-pit | Stone for building | Nonmetal mine | Small | 40 | 0 |
| 125 | Shandong | Jinan | Underground | Iron | Ferrous metal mine | Big | 783 | 0 |
| 126 | Shandong | Taian | Open-pit | Limestone | Nonmetal mine | Big | 115 | 0 |
