# Supplementary material for: Occupational Exposure to Silica Dust and Silicosis Risk in Chinese Noncoal Mines: Qualitative and Quantitative Risk Assessment
Source: JMIR Public Health Surveill. 2024 Sep 2;10:e56283. doi: 10.2196/56283 (PMC11406111; doi:10.2196/56283)
Supplement: Multimedia Appendix 3 [file publichealth_v10i1e56283_app3.doc]

**Table S3. The number of workers and dust exposure levels of the included 126 non-coal mines. No: Number; *M*: median; IQR: interquartile range.**

| Characteristics | No of workers (female) | Free silica content | | *M* of total silica dust concentration (IQR, mg/m^3^) | *M* of respirable silica dust concentration (IQR, mg/m^3^) |
| --- | --- | --- | --- | --- | --- |
|  |  | No of dust samples | *M* (IQR, %) |  |  |
| **Overall** | 29835 (1739) | 1566 | 27.6 (17.7-38.1) | 0.39 (0.18-0.73) | 0.15 (0.09-0.31) |
| **Mine category** |  |  |  |  |  |
| **Nonferrous metal mine** | 8635 (584) | 501 | 21.2 (17.0-30.5) | 0.36 (0.13-0.71) | 0.17 (0.06-0.31) |
| Gold | 1095 (26) | 123 | 26.3 (18.8-39.3) | 0.37 (0.15-0.92) | 0.14 (0.08-0.37) |
| Lead-zinc | 2349 (160) | 180 | 19.8 (14.8-29.4) | 0.31 (0.12-0.59) | 0.17 (0.05-0.30) |
| Copper | 4122 (390) | 117 | 17.2 (17.0-24.9) | 0.39 (0.11-0.52) | 0.15 (0.06-0.25) |
| Silver | 1051 (8) | 45 | 32.3 (24.6-36.5) | 0.73 (0.45-0.88) | 0.39 (0.24-0.53) |
| Antimony | 18 (0) | 36 | 16.7 (16.6-17.3) | 0.13 (0.09-0.36) | 0.04 (0.02-0.06) |
| **Ferrous metal mine (Only iron)** | 17116 (921) | 363 | 22.1 (15.1-28.4) | 0.24 (0.14-0.44) | 0.11 (0.06-0.19) |
| **Nonmetal mine** | 4084 (234) | 702^a^ | 36.9 (27.6-41.2) | 0.48 (0.31-0.95) | 0.16 (0.12-0.38) |
| Stone for building | 373 (4) | 285 | 32.1 (28.5-38.1) | 0.49 (0.35-1.42) | 0.16 (0.11-0.49) |
| Sandstone for glassmaking | 195 (23) | 105 | 38.1 (18.1-38.5) | 0.36 (0.20-0.51) | 0.14 (0.10-0.19) |
| Zeolite | 38 (4) | 45 | 26.7 (22.1-38.1) | 0.53 (0.48-1.24) | 0.35 (0.16-0.42) |
| Silica rock | 827 (45) | 45 | 83.0 (65.4-93.6) | 1.25 (0.36-1.67) | 0.72 (0.25-0.91) |
| Refractory clay | 66 (0) | 57 | 38.7 (37.2-39.2) | 0.33 (0.29-0.45) | 0.14 (0.12-0.16) |
| Sand for building | 65 (1) | 48 | 31.5 (20.0-33.6) | 0.62 (0.29-1.06) | 0.17 (0.10-0.36) |
| Jewel | 264 (11) | 18 | 54.7 (44.5-60.3) | 0.78 (0.52-1.54) | 0.28 (0.22-0.37) |
| Phosphorus | 496 (35) | 9 | 11.1 (10.7-17.2) | 0.03 (0.01-0.22) | 0.01 (0.01-0.12) |
| Fluorite | 407 (75) | 42 | 39.2 (38.0-16.6) | 0.41 (0.37-1.19) | 0.23 (0.16-0.43) |
| Dolomite | 21 (0) | 24 | 43.8 (42.8-44.8) | 0.77 (0.59-1.01) | 0.24 (0.19-0.32) |
| Kaolin and China clay | 1217 (36) | 18 | 39.5 (39.5-40.6) | 0.40 (0.32-0.53) | 0.22 (0.17-0.23) |
| Limestone | 115 (0) | 6 | 17.6 (17.5-17.7) | 0.14 (0.13-0.16) | 0.10 (0.09-0.10) |
| **Production scale** |  |  |  |  |  |
| Big | 19177 (1006) | 363 | 23.4 (15.1-29.0) | 0.23 (0.13-0.45) | 0.11 (0.05-0.17) |
| Middle | 7218 (523) | 303 | 27.8 (21.2-36.5) | 0.36 (0.22-0.79) | 0.16 (0.10-0.32) |
| Small | 3440 (210) | 900 | 31.7 (19.2-39.0) | 0.45 (0.24-0.89) | 0.18 (0.10-0.37) |
| **Mining method** |  |  |  |  |  |
| Underground | 19387 (1181) | 645 | 21.2 (17.0-28.4) | 0.32 (0.14-0.56) | 0.15 (0.07-0.28) |
| Open-pit | 10448 (558) | 921 | 32.1 (21.6-39.2) | 0.43 (0.24-0.86) | 0.15 (0.10-0.34) |

^a^A total of nine dust samples were excluded due to the reason: three respirable dust samples’ concentrations were greater than their corresponding total dust concentrations, so the other three bulk dust samples for free silica content determination were excluded.
