# Supplementary figures and images for: Occupational Exposure to Silica Dust and Silicosis Risk in Chinese Noncoal Mines: Qualitative and Quantitative Risk Assessment
Source: JMIR Public Health Surveill. 2024 Sep 2;10:e56283. doi: 10.2196/56283 (PMC11406111; doi:10.2196/56283)

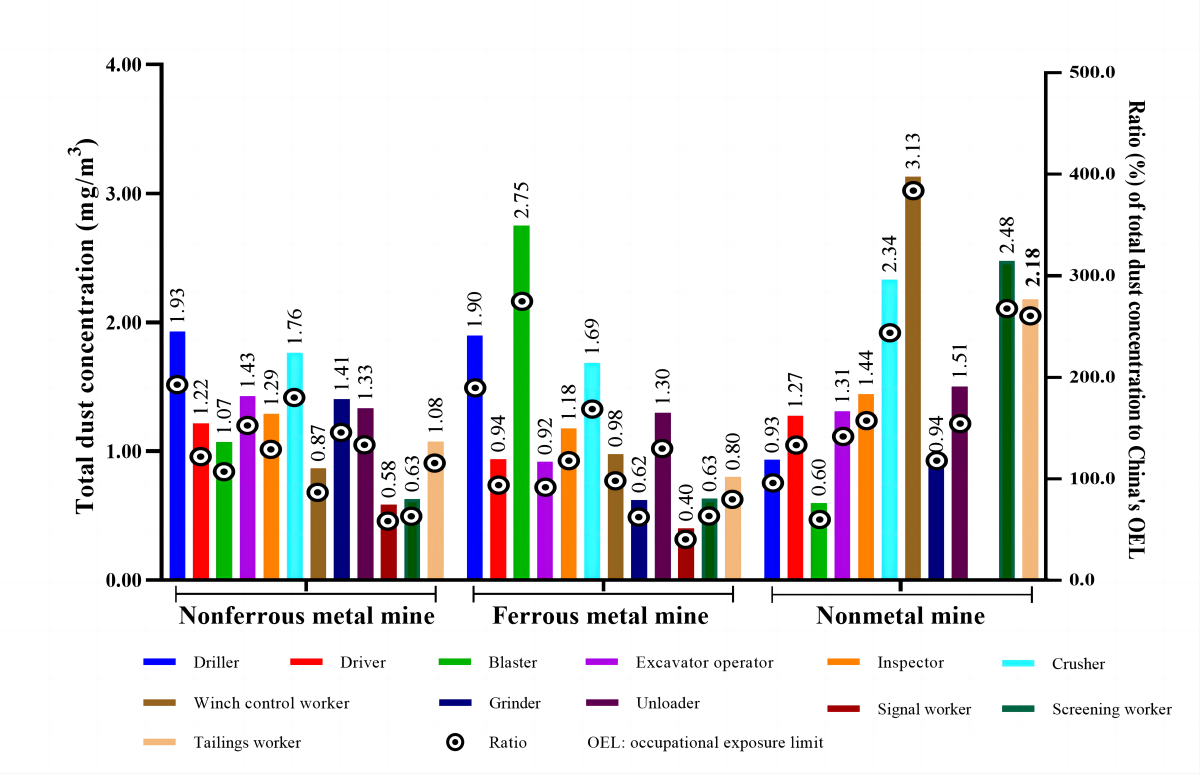

Supplement: Multimedia Appendix 4 [file publichealth_v10i1e56283_app4.png]

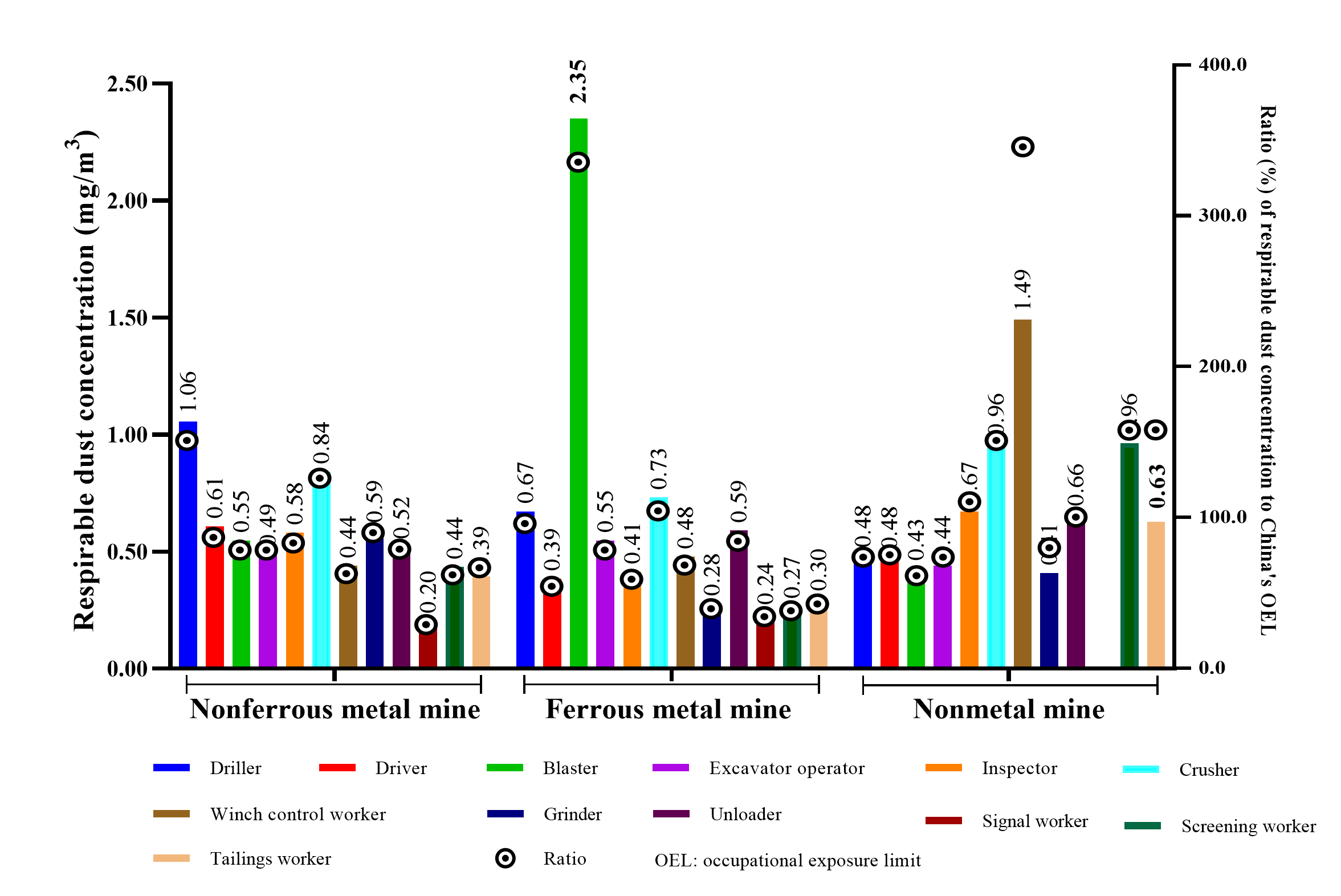

Supplement: Multimedia Appendix 5 [file publichealth_v10i1e56283_app5.png]
