# Supplementary material for: Occupational Exposure to Silica Dust and Silicosis Risk in Chinese Noncoal Mines: Qualitative and Quantitative Risk Assessment
Source: JMIR Public Health Surveill. 2024 Sep 2;10:e56283. doi: 10.2196/56283 (PMC11406111; doi:10.2196/56283)
Supplement: Multimedia Appendix 6 [file publichealth_v10i1e56283_app6.doc]

**Table S4. Under the exposure to total non-coal mine silica dust, the raw five-level risk assessment results of ICMM and INDEX methods. ICMM: International Mining and Metals Commission's risk rating table; INDEX: occupational hazard risk index.**

| **Characteristic** | **ICMM (%)** | | | | | **INDEX (%)** | | | | |
| --- | --- | --- | --- | --- | --- | --- | --- | --- | --- | --- |
|  | **Tolerable Risk** | **Potential Risk** | **High Risk** | **Very High Risk** | **Intolerable Risk** | **No Hazard** | **Mild Hazard** | **Moderate Hazard** | **Severe Hazard** | **Extreme Hazard** |
| **Overall** | 529 (35.3)^a^ | -^b^ | 3 (0.2) | 258 (17.2) | 710 (47.3) | 143 (9.5) | 401 (26.7) | 409 (27.3) | 332 (22.1) | 215 (14.3) |
| **Mine category** |  |  |  |  |  |  |  |  |  |  |
| Nonferrous metal mine | 195 (28.4) |  | 3 (0.4) | 204 (29.7) | 285 (41.5) | 19 (2.8) | 162 (23.6) | 205 (29.8) | 149 (21.7) | 152 (22.1) |
| Ferrous metal mine (Only iron) | 173 (48.5) |  |  | 38 (10.6) | 146 (40.9) | 48 (13.5) | 142 (39.8) | 92 (25.8) | 51 (14.3) | 24 (6.7) |
| Nonmetal mine | 161 (35.3) |  |  | 16 (3.5) | 279 (61.2) | 76 (16.7) | 97 (21.3) | 112 (24.6) | 132 (29.0) | 39 (8.6) |
| **Production scale** |  |  |  |  |  |  |  |  |  |  |
| Big | 193 (53.2) |  |  | 38 (10.5) | 132 (36.4) | 65 (17.9) | 138 (38.0) | 75 (20.7) | 68 (18.7) | 17 (4.7) |
| Middle | 94 (35.2) |  | 3 (1.1) | 24 (9.0) | 146 (54.7) | 8 (3.0) | 68 (25.5) | 84 (31.5) | 69 (25.8) | 38 (14.2) |
| Small | 242 (27.8) |  |  | 196 (22.5) | 432 (49.7) | 70 (8.1) | 195 (22.4) | 250 (28.7) | 195 (22.4) | 160 (18.4) |
| **Mining method** |  |  |  |  |  |  |  |  |  |  |
| Underground | 218 (37.3) |  |  | 26 (4.4) | 341 (58.3) | 83 (14.2) | 152 (26.0) | 157 (26.8) | 148 (25.3) | 45 (7.7) |
| Open-pit | 311 (34.0) |  | 3 (0.3) | 232 (25.4) | 369 (40.3) | 60 (6.6) | 249 (27.2) | 252 (27.5) | 184 (20.11 | 170 (18.6) |
| **Job** |  |  |  |  |  |  |  |  |  |  |
| Driller | 42 (30.4) |  |  | 12 (8.7) | 84 (60.9) | 14 (10.1) | 30 (21.7) | 36 (26.1) | 42 (30.4) | 16 (11.6) |
| Driver | 179 (35.5) |  |  | 187 (37.1) | 138 (27.4) | 21 (4.2) | 171 (33.9) | 199 (39.5) | 65 (12.9) | 48 (9.5) |
| Blaster | 12 (50.0) |  |  |  | 12 (50.0) | 3 (12.5) | 9 (37.5) | 3 (12.5) | 9 (37.5) |  |
| Excavator operator | 42 (36.8) |  |  | 9 (7.9) | 63 (55.3) | 6 (5.3) | 38 (33.3) | 34 (29.8) | 25 (21.9) | 11 (9.7) |
| Inspector | 62 (44.9) |  | 3 (2.2) | 12 (8.7) | 61 (44.2) | 35 (25.4) | 28 (20.3) | 19 (13.8) | 43 (31.2) | 13 (9.4) |
| Crusher | 55 (25.1) |  |  | 7 (3.2) | 157 (71.7) | 7 (3.2) | 36 (16.4) | 43 (19.6) | 67 (30.6) | 66 (30.1) |
| Winch control worker | 8 (24.2) |  |  |  | 25 (75.7) | 3 (9.1) | 5 (15.2) | 10 (30.3) | 3 (9.1) | 12 (36.4) |
| Grinder | 17 (37.8) |  |  | 3 (6.7) | 25 (55.6) | 4 (8.9) | 13 (28.9) | 13 (28.9) | 9 (20.0) | 6 (13.3) |
| Unloader | 61 (38.4) |  |  | 22 (13.8) | 76 (47.8) | 26 (16.4) | 42 (26.4) | 37 (23.3) | 42 (26.4) | 12 (7.6) |
| Packing worker | 3 (20.0) |  |  |  | 12 (80.0) |  | 3 (20.0) |  | 6 (40.0) | 6 (40.0) |
| Signal worker | 9 (75.0) |  |  | 3 (25.0) |  | 9 (75.0) | 1 (8.3) | 2 (16.7) |  |  |
| Screening worker | 23 (42.6) |  |  | 3 (5.6) | 28 (51.8) | 8 (14.8) | 14 (25.9) | 6 (11.1) | 10 (18.5) | 16 (29.6) |
| Tailings worker | 13 (31.0) |  |  |  | 29 (69.0) | 7 (16.7) | 8 (19.1) | 7 (16.7) | 11 (26.2) | 9 (21.4) |
| Stroker | 3 (100.0) |  |  |  |  |  | 3 (100.0) |  |  |  |

^a^There was no realted data in this part.
