# Supplementary material for: Occupational Exposure to Silica Dust and Silicosis Risk in Chinese Noncoal Mines: Qualitative and Quantitative Risk Assessment
Source: JMIR Public Health Surveill. 2024 Sep 2;10:e56283. doi: 10.2196/56283 (PMC11406111; doi:10.2196/56283)
Supplement: Multimedia Appendix 7 [file publichealth_v10i1e56283_app7.doc]

**Table S5. Under the exposure to respirable non-coal mine silica dust, the raw five-level risk assessment results of ICMM and INDEX methods. ICMM: International Mining and Metals Commission's risk rating table; INDEX: occupational hazard risk index.**

| **Characteristic** | **ICMM (%)** | | | | | **INDEX (%)** | | | | |
| --- | --- | --- | --- | --- | --- | --- | --- | --- | --- | --- |
|  | **Tolerable Risk** | **Potential Risk** | **High Risk** | **Very High Risk** | **Intolerable Risk** | **No Hazard** | **Mild Hazard** | **Moderate Hazard** | **Severe Hazard** | **Extreme Hazard** |
| **Overall** | 891 (57.2) | -^a^ | 3 (0.2) | 128 (8.2) | 535 (34.4) | 295 (19.0) | 550 (35.3) | 387 (24.9) | 215 (13.8) | 110 (7.1) |
| **Mine category** |  |  |  |  |  |  |  |  |  |  |
| Nonferrous metal mine | 398 (56.7) |  |  | 59 (8.4) | 245 (34.9) | 78 (11.1) | 241 (34.3) | 188 (26.8) | 119 (17.0) | 76 (10.8) |
| Ferrous metal mine (Only iron) | 239 (67.0) |  | 3 (0.8) | 45 (12.6) | 70 (19.6) | 92 (25.8) | 184 (51.5) | 52 (14.6) | 27 (7.6) | 2 (0.6) |
| Nonmetal mine | 254 (51.0) |  |  | 24 (4.8) | 220 (44.2) | 125 (25.1) | 125 (25.1) | 147 (29.5) | 69 (13.9) | 32 (6.4) |
| **Production scale** |  |  |  |  |  |  |  |  |  |  |
| Big | 257 (70.8) |  | 3 (0.8) | 23 (6.3) | 80 (22.0) | 98 (27.0) | 190 (52.3) | 45 (12.4) | 25 (6.9) | 3 (1.4) |
| Middle | 159 (53.5) |  |  | 25 (8.4) | 113 (38.1) | 38 (12.8) | 94 (31.7) | 99 (33.3) | 39 (13.1) | 27 (9.1) |
| Small | 475 (53.0) |  |  | 80 (8.9) | 342 (38.1) | 159 (17.7) | 266 (29.7) | 243 (27.1) | 151 (16.8) | 78 (8.7) |
| **Mining method** |  |  |  |  |  |  |  |  |  |  |
| Underground | 338 (52.7) |  |  | 45 (7.0) | 259 (40.3) | 138 (21.5) | 208 (32.4) | 183 (28.5) | 82 (12.8) | 31 (4.8) |
| Open-pit | 553 (60.4) |  | 3 (0.3) | 83 (9.1) | 276 (30.2) | 157 (17.2) | 342 (37.4) | 204 (22.3) | 133 (14.5) | 79 (8.6) |
| **Job** |  |  |  |  |  |  |  |  |  |  |
| Driller | 47 (34.1) |  |  | 26 (18.8) | 65 (47.1) | 24 (17.4) | 37 (26.8) | 39 (28.3) | 32 (23.2) | 6 (4.4) |
| Driver | 372 (72.9) |  |  | 40 (7.8) | 98 (19.2) | 88 (17.3) | 246 (48.2) | 111 (21.8) | 30 (5.9) | 35 (6.9) |
| Blaster | 14 (58.3) |  |  | 1 (4.2) | 9 (37.5) | 5 (20.8) | 7 (29.2) | 1 (4.2) | 11 (45.8) |  |
| Excavator operator | 96 (74.4) |  |  | 3 (2.3) | 30 (23.3) | 22 (17.1) | 56 (43.4) | 31 (24.0) | 11 (8.5) | 9 (7.0) |
| Inspector | 88 (62.4) |  |  | 15 (10.6) | 38 (27.0) | 51 (36.2) | 38 (27.0) | 34 (24.1) | 15 (10.6) | 3 (2.1) |
| Crusher | 66 (29.3) |  |  | 21 (9.3) | 138 (61.4) | 15 (6.7) | 59 (26.2) | 73 (32.4) | 56 (24.9) | 22 (9.8) |
| Winch control worker | 12 (33.3) |  |  |  | 24 (66.7) | 3 (8.3) | 8 (22.2) | 10 (27.8) | 3 (8.3) | 12 (33.3) |
| Grinder | 34 (59.7) |  |  |  | 23 (40.4) | 16 (28.1) | 13 (22.8) | 13 (22.8) | 12 (21.1) | 3 (5.3) |
| Unloader | 101 (59.0) |  | 3 (1.8) | 14 (8.2) | 53 (31.0) | 40 (23.4) | 60 (35.1) | 48 (28.1) | 15 (8.8) | 8 (4.7) |
| Packing worker | 3 (20.0) |  |  |  | 12 (80.0) |  | 3 (20.0) | 2 (13.3) | 4 (26.7) | 6 (40.0) |
| Signal worker | 10 (83.3) |  |  | 2 (16.7) |  | 9 (75.0) | 3 (25.0) |  |  |  |
| Screening worker | 27 (50.0) |  |  | 6 (11.1) | 21 (38.9) | 10 (18.5) | 13 (24.1) | 13 (24.4) | 18 (33.3) |  |
| Tailings worker | 18 (42.9) |  |  |  | 24 (57.1) | 11 (26.2) | 5 (11.9) | 12 (28.6) | 8 (19.1) | 6 (14.3) |
| Stroker | 3 (100.0) |  |  |  |  | 1 (33.3) | 2 (66.7) |  |  |  |

^a^There was no realted data in this part.
